# *Ddhd1* knockout mouse as a model of locomotive and physiological abnormality in familial spastic paraplegia

**DOI:** 10.1042/BSR20204171

**Published:** 2021-02-26

**Authors:** Takuya Morikawa, Hiroaki Ohishi, Kengo Kosaka, Tomofumi Shimojo, Akihiro Nagano, Itsuki Taniguchi, Ryuta Fujioka, Kosei Moriyama, Motoko Unoki, Masatomo Takahashi, Motonao Nakao, Yoshihiro Izumi, Takeshi Bamba, Hiroyuki Sasaki, Shiroh Miura, Hiroki Shibata

**Affiliations:** 1 Division of Genomics, Medical Institute of Bioregulation, Kyushu University, 3-1-1, Maidashi, Higashi-ku, Fukuoka, Japan; 2 Division of Epigenomics and Development Medical Institute of Bioregulation, Kyushu University, 3-1-1, Maidashi, Higashi-ku, Fukuoka, Japan; 3 Department of Food and Nutrition, Beppu University Junior College, 82, Kitaishigaki, Beppu, Oita, Japan; 4 Department of Nutritional Sciences, Nakamura Gakuen University, 5-7-1, Befu, Zyonan-ku, Fukuoka, Japan; 5 Division of Metabolomics, Medical Institute of Bioregulation, Kyushu University, 3-1-1, Maidashi, Higashi-ku, Fukuoka, Japan; 6 Department of Neurology and Geriatric Medicine, Ehime University Graduate School of Medicine, 454, Shitsukawa, Toon, Ehime, Japan

**Keywords:** DDHD1, foot–base angle (FBA), lysophosphatidylinositol (LPI), PLA1, Spastic paraplegia (SPG)

## Abstract

We have previously reported a novel homozygous 4-bp deletion in *DDHD1* as the responsible variant for spastic paraplegia type 28 (SPG28; OMIM#609340). The variant causes a frameshift, resulting in a functionally null allele in the patient. *DDHD1* encodes phospholipase A_1_ (PLA_1_) catalyzing phosphatidylinositol to lysophosphatidylinositol (LPI). To clarify the pathogenic mechanism of SPG28, we established *Ddhd1* knockout mice (*Ddhd1*[−/−]) carrying a 5-bp deletion in *Ddhd1*, resulting in a premature termination of translation at a position similar to that of the patient. We observed a significant decrease in foot–base angle (FBA) in aged *Ddhd1*(−/−) (24 months of age) and a significant decrease in LPI 20:4 (*sn*-2) in *Ddhd1*(−/−) cerebra (26 months of age). These changes in FBA were not observed in 14 months of age. We also observed significant changes of expression levels of 22 genes in the *Ddhd1*(−/−) cerebra (26 months of age). Gene Ontology (GO) terms relating to the nervous system and cell–cell communications were significantly enriched. We conclude that the reduced signaling of LPI 20:4 (*sn*-2) by PLA_1_ dysfunction is responsible for the locomotive abnormality in SPG28, further suggesting that the reduction of downstream signaling such as GPR55 which is agonized by LPI is involved in the pathogenesis of SPG28.

## Introduction

Spastic paraplegias (SPGs) are neurological disorders characterized by spasticity and gait disturbance. Abnormalities of the pyramidal tract are known to be a hallmark of this disorder. More than 60 types of SPGs have been reported to be genetically distinct [1]. SPG type 28 (SPG28) is an autosomal recessive SPG caused by mutations in the gene encoding DDHD domain-containing protein 1 (*DDHD1*) also known as phospholipase A_1_ (PLA_1_) [1–5]. We have previously identified a novel homozygous 4-bp deletion (c.914_917delGTAA, p.Ser^305^Ilefs*2) in exon 2 of the *DDHD1* gene as the variant responsible for SPG28 (OMIM#609324) [6]. Phospholipase A is known to catalyze phosphatidylinositol (PI) and phosphatidic acid (PA) to lysophosphatidylinositol (LPI) and lysophosphatidic acid (LPA), respectively. There are two kinds of phospholipase A; PLA_1_ such as DDHD1, hydrolyzes the *sn*-1 ester bond of PI, and phospholipase A_2_ (PLA_2_) hydrolyzes the *sn*-2 ester bond of PI [7]. The intracellular PLA_1_ protein family is characterized by the presence of a short lipase active-site sequence and a C-terminal DDHD domain. *DDHD1* is known to be highly expressed in the brain and testes and its dysfunction causes neurodegeneration with brain iron accumulation (NBIA) as well as SPG phenotypes [8–9].

Animal models are useful in clarification the pathogenic mechanism and potential therapy. There are two previous studies of *Ddhd1* knockout (KO) mice [8,10]. One has reported impaired movement of sperm and abnormal mitochondrial morphology with no description of abnormal mobility [8]. The other study has reported a significant increase in PI 18:1/20:4 and a significant decrease in LPI 20:4 in cerebra of their *Ddhd1* KO mice. The authors, however have not described any abnormal mobility in 6-month-old *Ddhd1* KO mice [10]. SPG onset in humans varies at ages from 0 to 70 years and deteriorates in aged patients, suggesting that the mice examined in these previous studies might have been too young for the authors to examine SPG phenotypes [2]. To clarify the pathogenic mechanism of SPG28, we established *Ddhd1*(−/−) and perform behavioral analyses (14 and 24 months of age). We also performed RNA sequencing and lipidome analysis on sufficiently aged mice (26 months of age).

## Results

### Establishment of *Ddhd1* KO mice

**Figure 1:**
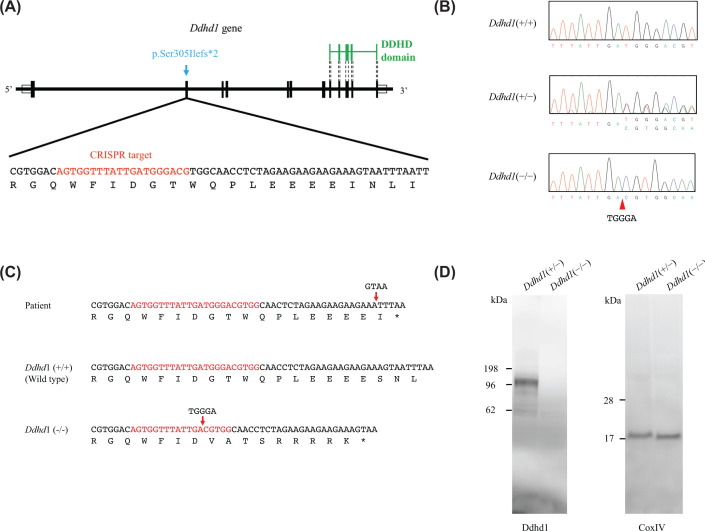
*Ddhd1* knockout mouse carrying a 5-bp deletion (**A**) Gene structure of *Ddhd1*. Vertical ticks and white boxes represent coding and noncoding regions, respectively. Green lines indicate the DDHD domain. DNA sequences in the part of exon 2 are shown below. CRISPR target sequences are indicated by red letters. The variant we identified is shown as blue. (**B**) Sequencing electrogram of the wild type, *Ddhd1*(+/−), and *Ddhd1*(−/−). The arrowhead indicates 5-bp deletion. (**C**) Mice harboring 5-bp deletion causing a frameshift in *Ddhd1* and the premature termination at the position of slight upstream compared with the variant in the patient. CRISPR target regions are shown in red. (**D**) Western blotting of cerebral tissues detected by an anti-DDHD1 antibody and an anti-COXIV antibody. An anti-COXIV antibody was used as a loading control.

We generated *Ddhd1* KO mice using the CRISPR/Cas9 system according to Yang et al. (2014) [11] (Figure 1A). We identified four kinds of small indels that are expected to cause frameshift and premature termination at *Ddhd1*. We selected a mouse harboring 5-bp deletion in exon 2 since the resulting amino acid sequence is the closest to the variant found in SPG28 patients among some kinds of indels created by CRISPR/Cas9 (Figure 1B,C) [6]. To remove off-target sites from the mice harboring 5-bp deletion, we performed one-generation backcross with C57BL/6J. By crossing the F_1_ mice heterozygous for the 5-bp deletion, we established the *Ddhd1*(−/−) strain as the *Ddhd1* KO mice carrying the premature termination at a very similar position with the variant we identified in the original patient. We also confirmed the absence of DDHD1 protein signal in *Ddhd1*(−/−) mice by Western blotting (Figure 1D). Full-length blots are presented in Supplementary Figure S1. In the following analyses, we use heterozygous KO mice (*Dhdh1*[+/−]) as a control for homozygous KO mice (*Ddhd1*[−/−]).

### Foot–base angle analysis

**Figure 2:**
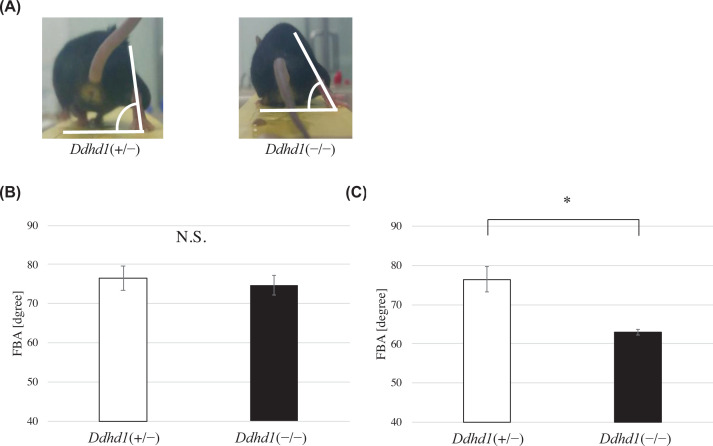
Foot-base angle (FBA) analysis (**A**) Single video frames of a *Ddhd1*(+/−) and *Ddhd1*(−/−) mice walking on the bridge are shown as examples. FBA at toe-off position is indicated by white lines. (**B**) The FBA of mice at the 14 months of age. There were no significant differences between genotypes (*n*=3). (**C**) The FBA of mice at 24 months of age. *Ddhd1*(−/−) (*n*=3) showed a significant decrease in FBA compared with *Ddhd1*(+/−) (*n*=2). Same animals at different ages were used except one mouse of *Ddhd1*(+/−) which died after the examination at 14 months of age. Error bars represent the mean ± SD. For *Ddhd1*(+/−) versus *Ddhd*1(−/−), *: *P*=5.0 × 10^−3^ N.S., not significant. All data were analyzed using a two-tailed Student’s *t* test.

We examined the behavioral mobility of 14- and 24-month-old *Ddhd1*(+/−) and *Ddhd1*(−/−) by measuring foot–base angle (FBA), which is an established method to evaluate the weakness of hindlimbs in mice (Figure 2A) [12,13]. At 14 months of age, FBA did not show significant difference between *Ddhd1*(+/−) and *Ddhd1*(−/−) (*P*=0.48) and there were also no significant differences between any of the individuals (*n*=3) (Figure 2B, Supplementary Table S1A and Figure S2A). At 24 months of age, *Ddhd1*(−/−) (*n*=3) showed significant decrease in FBA compared with *Ddhd1*(+/−) (*n*=2) (*P*=5.0 × 10^−3^) (Figure 2C). In individual comparison by Tukey’s test, we consistently observed significant decrease in FBA in all combinations of *Ddhd1*(+/−) and *Ddhd1*(−/−) (*P*<1.1 × 10^−2^) (Supplementary Figure S2B and Table S1B). The absence of the FBA phenotype in 14-month-old *Ddhd1*(−/−) mice suggested that it takes at least more than 14 months to manifest the FBA phenotypes in *Ddhd1*(−/−) mice.

### Lipidome analysis

**Figure 3:**
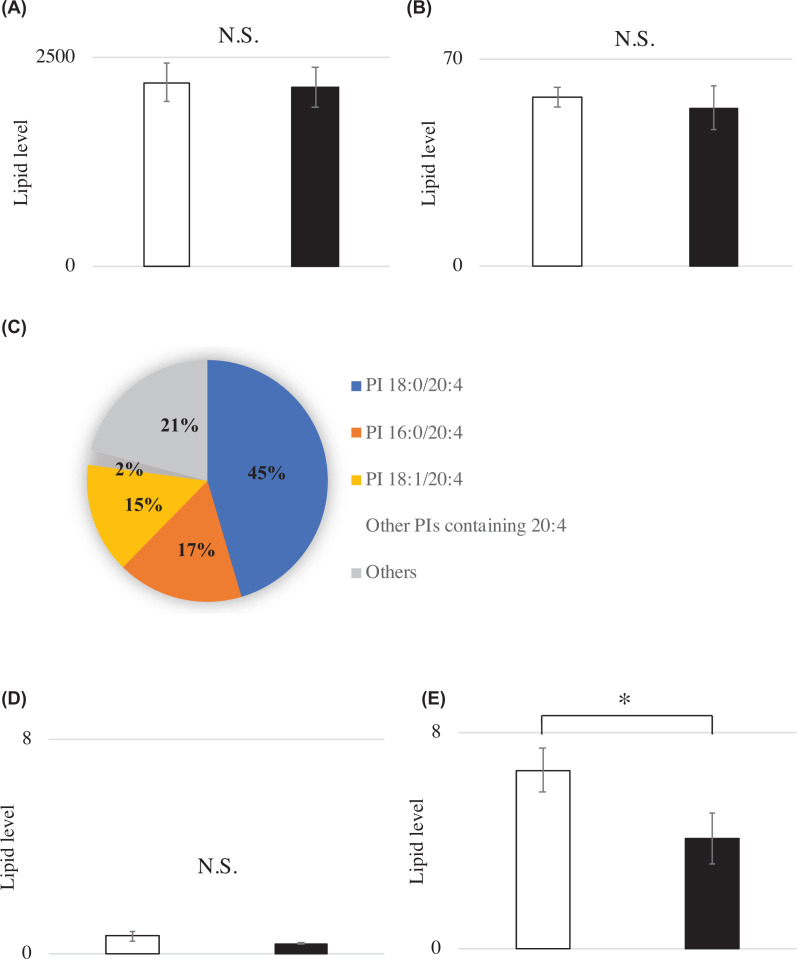
Lipidome analysis (**A**,**B**) Total quantity of (A) PI and (B) LPI in mouse cerebra. (**C**) Pie charts of the PIs identified in mouse cerebra. (**D**,**E**) The quantity of LPI 20:4 specified by the digested positions. (D) LPI 20:4 (*sn*-1) catalyzed by PLA_2_ and (E) LPI 20:4 (*sn*-2) catalyzed by PLA_1_. *Ddhd1*(+/−) and *Ddhd1*(−/−) are shown in opened and filled columns, respectively. The unit of the vertical axis is pmol/mg. Error bars represent the mean ± SD. For *Ddhd1*(+/−) (*n*=2) versus *Ddhd*1(−/−) (*n*=2), *: *P*<0.05. N.S., not significant. All data were analyzed using a two-tailed Student’s t test.

We measured two kinds of metabolites of DDHD1, PIs and LPIs in cerebra from *Ddhd1*(+/−) and *Ddhd1*(−/−) mice (*n*=2 for each) using supercritical fluid chromatography triple–quadrupole mass spectrometry (SFC/QqQMS) [14]. We identified 49 kinds of PIs and 6 kinds of LPIs in mouse cerebra (Supplementary Table S2). In spite of the dysfunction of *Ddhd1*, the *Ddhd1*(−/−) mice did not show a significant difference in total quantities of PI (Figure 3A) and LPI (Figure 3B). We also observed that the majority of PIs contain arachidonic acid (20:4), which accounted for approximately 79% of the total amount of PIs in mouse cerebra (Figure 3C). Of the 49 kinds of PIs, we identified 14 containing arachidonic acid. By comparing quantities of PI containing arachidonic acid in *Ddhd1*(+/−) with those in *Ddhd1*(−/−), we observed a significant increase in PI 20:4/20:4 in *Ddhd1*(−/−) (Supplementary Figure S3). Although LPI 20:4 (*sn*-1), the metabolite of PLA_2_, did not show a difference between *Ddhd1*(+/−) and *Ddhd1*(−/−) (Figure 3D), LPI 20:4 (*sn*-2), the metabolite excised from PIs containing arachidonic acid by PLA_1_, was significantly decreased in *Ddhd1*(−/−) (Figure 3E). This significant decrease in LPI (*sn*-2) was not observed in the cerebella of *Ddhd1*(−/−) (Supplementary Figure S4). Retention times of LPI 20:4 (*sn*-1) and LPI 20:4 (*sn*-2) were 19.55 and 19.70 min, respectively (Supplementary Table S2 and Figure S5). In contrast, neither PAs nor LPAs were significantly changed in cerebra of *Ddhd1*(−/−) (Supplementary Table S3).

### RNA sequencing and gene ontology enrichment analysis 

**Figure 4:**
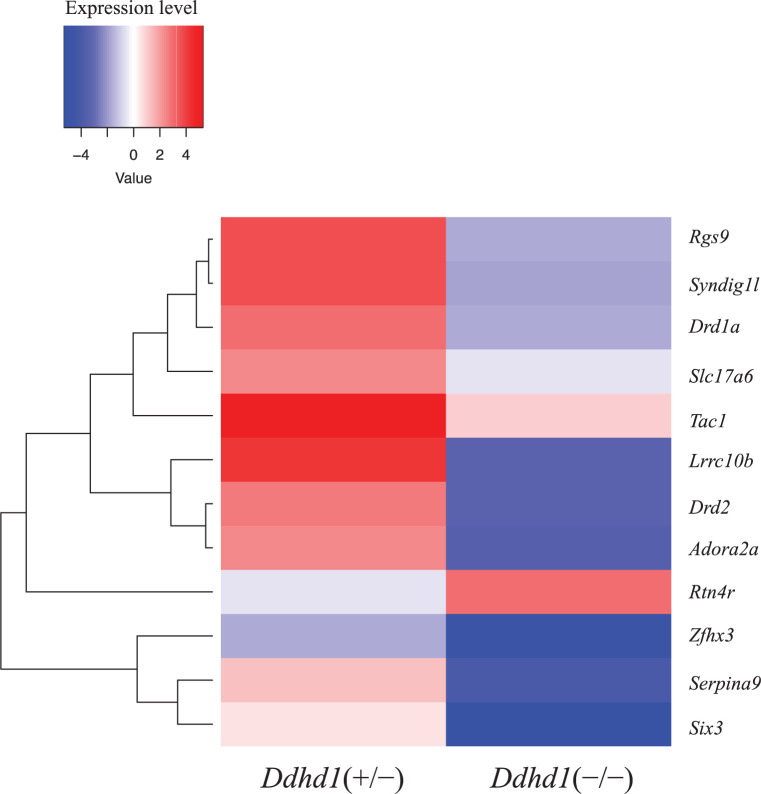
DEGs in *Ddhd1*(−/−) Heatmaps of DEGs identified by RNA sequencing of *Ddhd1*(+/−) (*n*=2) and *Ddhd1*(−/−) (*n*=2) at 26 months of age. The left lane shows *Ddhd1*(+/−); the right lane shows *Ddhd1*(−/−). Gene names are shown in vertical lines based on hierarchical clustering. The colored scale bar on the top left side indicates relative expression value where −4 and 4 represent the down- and up-regulation of each gene, respectively. Expression levels were calculated as log_10_ (FPKM) − average FPKM. Genes that showed that FPKM was zero in either *Ddhd1*(+/−) or *Ddhd1*(−/−) were excluded from the heatmap. Abbreviation: FPKM, fragments per kilobase of exon per million fragments mapped.

We sequenced total RNA extracted from the cerebra of two *Ddhd1*(+/−) and two *Ddhd1*(−/−). We obtained an average of 30609158 reads per mouse used in the present study. By comparing gene expression levels of *Ddhd1*(+/−) with that of *Ddhd1*(−/−), we identified 22 differentially expressed genes (DEGs), including three up-regulated genes and 19 down-regulated genes in *Ddhd1*(−/−) (Figure 4 and Supplementary Table S4). Using gene ontology enrichment analysis (GEA), we identified two annotation clusters which include significant gene ontology (GO) terms (Benjamini probabilities < 0.05) as shown in Table 1. We identified significantly enriched terms related to the nervous system as GO:0007268 (synaptic transmission), GO:0051966 (regulation of synaptic transmission, glutamatergic), GO:0019226 (transmission of nerve impulse), and GO:0007270 (nerve–nerve synaptic transmission). GO terms related to cell–cell communication, such as GO:0007267 (cell–cell signaling), were also enriched as some of significant GO terms (Table 1). We have previously reported that the decrease in *DDHD1* mRNA expression level in the peripheral blood of SPG28 patients compared with unrelated control due to the nonsense-mediated decay [6]. Although *Ddhd1* was not selected as a significant DEG in the current RNA-seq analysis, we confirmed the decrease in the *Ddhd1* transcription level by approximately 55% in the *Ddhd1*(−/−) mice cerebra (Supplementary Table S5). Among the DEGs, we examined the expression of *Rtn4r* by real-time quantitative PCR (RT-qPCR) analysis. We observed significant increase in *Rtn4r* and *Adra2a* expression in *Ddhd1*(−/−) at the 12 months of age consistent with the result of RNA sequencing (Supplementary Figure S6).

**Table 1:** GO terms enriched in *Ddhd1* KO mice

Annotation Cluster 1	Enrichment score: 2.53			
GO	Term	Benfamini probability	Number of genes	Genes
0007267*	Cell–cell signaling	8.3 × 10^−3^	6	*Adora2a, Drd1a, Drd2*, Six3, Slc17a6, Tac1
0010648*	Negative regulation of cell communication	2.2 × 10^−2^	4	*Drd1a, Drd2, Rgs9, Six3*

Annotation Cluster 2	Enrichment score: 2.22			
GO	Term	Benfamini probability	Number of genes	Genes
0007276*	Cell–cell signaling	8.3 × 10^−3^	6	*Adora2a, Drd1a, Drd2, Six3, Slc17a6, Tac1*
0007268*	Synaptic transmission	1.2 × 10^−2^	5	*Adora2a, Drd1a, Drd2, Slc17a6, Tac1*
0007626	Locomotory behavior	1.3 × 10^−2^	5	*Adora2a, Ccl21a, Ccl21b, Drd1a, Drd2*
0051966*	Regulation of synaptic transmission, glutamatergic	1.5 × 10^−2^	3	*Adora2a, Drd1a, Drd2*
0001975	Response to amphetamine	1.5 × 10^−2^	3	*Adora2a, Drd1a, Drd2*
0019226*	Transmission of nerve impulse	1.6 × 10^−2^	5	*Adora2a, Drd1a, Drd2, Slc17a6, Tac1*
0014075	Regulation of locomotion	1.8 × 10^−2^	4	*Adora2a, Ccl21a, Drd1a, Drd2*
0010243	Response to organic nitrogen	1.9 × 10^−2^	3	*Adora2a, Drd1a, Drd2*
0040012	Response to amine stimulus	1.9 × 10^−2^	3	*Adora2a, Drd1a, Drd2*
0007270*	Nerve–nerve synaptic transmission	2.9 × 10^−2^	3	*Adora2a, Drd1a, Drd2*
0043279	Response to alkaloid	3.0 × 10^−2^	3	*Adora2a, Drd1a, Drd2*
0051294	Positive regulation of multicellular organismal process	4.1 × 10^−2^	4	*Adora2a, Drd1a, Drd2, Gh*
0014070	Response to organic cyclic substance	4.4 × 10^−2^	3	*Adora2a, Drd1a, Drd2*
0009719	Response to endogenenous stimulus	4.5 × 10^−2^	4	*Adora2a, Drd1a, Drd2, Gh*
0007610	Behavior	4.7 × 10^−2^	5	*Adora2a, Ccl21a, Ccl21b, Drd1a, Drd2*
0048167*	Regulation of synaptic plasticity	4.7 × 10^−2^	3	*Adora2a, Drd1a, Drd2*

Some GO terms significantly enriched in DEGs in *Ddhd1*(−/−) mouse. Two annotation clusters included significant GO terms (Benjamini probability < 0.05). GO terms related to nervous system or cell–cell communications are marked with asterisks (*).

### Immunohistochemistry

**Figure 5:**
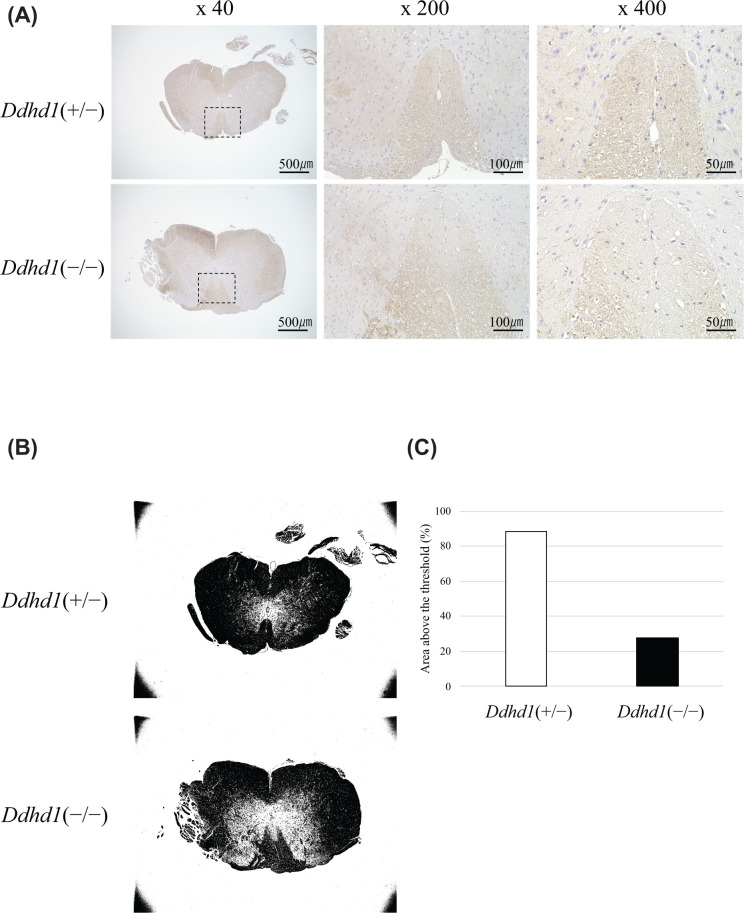
Immunohistochemistry of mouse spinal cords (**A**) We stained spinal cords obtained from 6-month-old *Ddhd1*(+/−) and *Ddhd1*(−/−) with anti-NF antibody. The top shows the ventral side and the bottom shows the dorsal side. The pyramidal tracts were photographed at ×40, ×200 and ×400 magnification. The black dashed lines in the ×40 images show the field of ×200 images. (**B**) Areas stained darker than the threshold are shown in black. Images photographed with the ×40 magnification were analyzed using the matched threshold value. (**C**) Percentage of areas in the pyramidal tract that are stained above the threshold.

We examined neurofilament (NF) protein expression as an axonal marker in spinal of mice at the 6 months of age. While there was no apparent morphological difference between *Ddhd1*(−/−) and *Ddhd1*(+/−), we observed remarkable decrease in staining of NF in the pyramidal tract of *Ddhd1*(−/−) compared with *Ddhd1*(+/−) (Figure 5A–C). This result indicates axonal decrease in the pyramidal tract in *Ddhd1*(−/−) at 6 months of age.

## Discussion

In the present study, we observed that gait disturbance in SPG patients was partially replicated in our *Ddhd1* KO mice, *Ddhd1*(−/−) at 24 months of age. Although there are two previous reports of *Ddhd1* KO mice [8,10], neither has reported locomotive abnormality. This discrepancy can be attributed to the difference in ages of mice examined. In one of the previous studies, locomotive phenotypes of *Ddhd1* KO mice were examined up to 6 months of age [10], while we examined FBA in 24 months of age, which are the oldest *Ddhd1* KO mice ever examined for their locomotion. Consistently we failed to observe the FBA phenotype in younger mice at the 14 months of age. This suggests that SPG phenotypes takes long time to manifest such as at least more than 14 months for the FBA phenotypes in *Ddhd1* KO mice. Fourteen months of age in mice is equivalent to forties in humans [15]. It is consistent with the observation that the age of SPG onset in human is known to be variable and sometimes to be seventies [2]. Therefore, even longer observation (>24 months) can be helpful to detect the SPG phenotypes in milder SPG mice. It is quite possible that abnormal locomotion could have been observed in other *Ddhd1* KO mice previously reported through the long-term observation. In addition, examination of the expression levels of genes involved in the LPI metabolism pathway in different ages may be helpful to clarify the mechanism of the deterioration of the phenotype along with aging.

The FBA test was originally established as an approach to assess muscle function in mice that had experienced femoral nerve damage [16]. Even though FBA is one of the simplest methods to quantitate locomotive activity without requiring special devices, it has not been applied to the examination of *Ddhd1* KO mice. Two strains of SPG model mice (subtypes SPG15 and SPG31) have also been reported to show significant decreases in their FBA [12,13], suggesting that FBA is an easy and reliable method in quantitative phenotyping of locomotion. From these studies, SPG15 and SPG31 model mice have observed symptoms by measuring FBA at 12 and 4 months, respectively. In contrast, we showed SPG28 takes much longer time to manifest the symptoms. As ages of SPG onset is known to be variable in humans, it is reasonable that ages of onset in SPG model mice are also variable among the types of SPGs.

Although gait disturbance is the common symptom in spinocerebellar ataxia (SCA) as well as in SPGs [23], their signs can be clearly distinguished by neurological examination. DDHD1 proteins are highly expressed in the cerebella as well as in the cerebra (Supplementary Figure S7). However, our previous neurological examination has confirmed no signs of cerebellar ataxia in the SPG28 patient in the original pedigree [6], indicating there is no involvement of cerebellum in the gait disturbance we observed in *Ddhd1* KO mice. Interestingly, the amount of LPI 20:4 (*sn*-2) decreased in the cerebra, but did not significantly change in the cerebella (*P*=0.09) (Supplementary Figure S4). While hindpaws of SCA model mice have been reported to be slipped off of the beam [24], we did not observe such slip-off in our *Ddhd1* KO mice during beam-walk test (data not shown). These data suggest that symptoms of gait disturbance is caused by a decrease in LPI in the cerebra, not in the cerebella.

In the present study we observed significant differences were observed in multiple PIs such as PI 16:0/20:4, PI 16:1/20:4, PI 18:1/20:4 and PI 20:4/20:4 in *Ddhd1* KO mice, while a previous study has reported only the increase in PI 18:1/20:4 [10]. This discrepancy can also be explained by the difference of examined mice (26 months in our study and 1.5–3 months in [10]). Although the previous study has reported the decrease in LPI 20:4 in *Ddhd1* KO mice [10], the researchers examined only total amount of LPIs without distinguishing their components, such as LPI 20:4 (*sn*-1) or LPI 20:4 (*sn*-2). In the current study, we observed the specific decrease in LPI 20:4 (*sn*-2) rather than that of LPI 20:4 (*sn*-1) in *Ddhd1*(−/−) by distinguishing isoforms of LPIs using SFC. Our result suggests that the decrease in total LPI 20:4 observed in previous study is due to a decrease in LPI 20:4 (*sn*-2), not LPI 20:4 (*sn*-1). LPIs are known to be endogenous agonists of GPR55, which might be involved in the regulation of axon growth and synaptic formation [17–22]. This observation suggests that the reduction in LPI 20:4 (*sn*-2) suppresses the signaling of the GPR55 and triggers abnormal axonal extension and incomplete neural circuits, eventually resulting in abnormal locomotion. To further examine the pathogenic mechanism, it is necessary to perform proteome analyses and functional analyses of neural cell formation in *Ddhd1*(−/−).

PA consumption and LPA production by the PA-PLA_1_ activity have been suggested to play an important role for mitochondrial fission [10]. However, our lipidome analyses observed no significant changes of PAs nor LPAs amount in *Ddhd1*(−/−) (Supplementary Table S3), suggesting that mitochondrial phenotypes may be associated with specific types of PIs and LPIs rather than PAs and LPAs.

In our current analysis, only three genes show significantly increased expression in cerebral tissues of *Ddhd1*(−/−), although we did not directly examine their protein expression levels. One of these, *Rtn4r*, is notable since it is known to mediate axonal growth inhibition and is involved in the regulation and plasticity of the adult central nervous system [25]. Since Rtn4r is known to activate RhoA, which is a downstream molecule of the GPR55 signaling, the increased expression of *Rtn4r* is possibly a compensatory feedback to recover the abnormal deactivation of GPR55 caused by *Ddhd1* dysfunction [26].

Interestingly, three genes encoding G protein-coupled receptors (GPCRs) were listed in DEGs, *Adra2a, Drd1a* and *Drd2* (Table 1 and Supplementary Table S4). Although these GPCRs do not interact with LPIs, the decrease in GPR55 signaling may disturb intracellular GTP metabolism, resulting in decreased expression of these GPCR genes.

We observed axonal degradation in the pyramidal tract in *Ddhd1*(−/−) at 6 months of age, which is much younger than the age we observed the weakness in hindlimbs by FBA analysis (24 months) (Figure 5). Therefore, axonal degradation in the pyramidal tract is likely to be one of the preclinical neurological changes preceding the onset of SPG phenotype. These results suggest that our *Ddhd1* KO mice at the age of 6 months are preclinical stage of SPG28 only showing a decrease in the number of axons.

*DDHD2*, another gene encoding PLA_1_, is also known to be responsible for SPG54 [27,28]. In addition, two other genes involved in the LPI metabolism pathway, *NTE* and *CYP2U1*, are also known to be responsible for SPG39 and SPG56, respectively [1,3]. Therefore, the disruption of the normal metabolism of LPIs is likely to be the common pathogenic mechanism shared by multiple SPGs, such as SPG28, SPG39, SPG54, and SPG56, suggesting the potential common drug for multiple SPGs.

SPG model mice reported here provide two major potential applications. One is the fine phenotyping of the disease progression. Physiological changes observed in the model mice provide seeds of biomarkers at the subclinical stages of SPG. The second is the platform for the development of therapeutic drugs. Candidate molecules can be deduced from the molecules connected to the DEGs identified in *Ddhd1*(−/−). Model mice can be examined at arbitrary ages so that the disease progression, the drug effects and the kinetics of candidate biomarkers can be analyzed through chronological snapshots.

## Materials and methods

### Animals

B6C3F1 and ICR mice were obtained from Kyudo company (Saga, Japan), and C57BL/6J mice were obtained from Charles River Laboratories Japan (Yokohama, Japan). They were fed a standard pellet diet (CLEA Japan, Inc., Tokyo, Japan) and filtered water. The animals were kept under condition of a 12:12-h light:dark cycle. All animal experiments were performed in Medical Institute of Bioregulation, Kyushu University.

### CRISPR construct generation

px330-U6-Chimeric_BB-CBh-hSpCas9 plasmid vectors harboring the cDNA sequence encoding *Streptococcus pyogenes* Cas9 (hSp-Cas9) and AmpR was purchased from Addgene (catalog 42230) [29]. Guide sequence oligonucleotides Mouse_CRISPR_target_S and Mouse_CRISPR_target_AS are shown in Supplementary Table S6. Oligonucleotides were annealed (95°C for 10 min followed by cooling down at room temperature for 30 min) and cloned in the px330 vector, which was digested with *BbsI*. Constructs were introduced into competent DH5α cells. The colonies harboring relevant constructs were inoculated into 5 ml Luria–Bertani medium and cultured overnight. Plasmid DNA was extracted from the culture using a Plasmid Maxi kit (#12165, Qiagen, Hilden, Germany).

### Microinjection

Pregnant mare serum gonadotropin and human chorionic gonadotropin were injected into B6C3H female mice at 48-h intervals. Injected females were then housed with C57BL/6 male mice. Fertilized one-celled stage embryos were collected from oviducts. We performed zygote injection according to Yang et al. (2014) [12]. We held a zygote using a holding pipette, inserted the injection pipette into the zygote without breaking the oolemma, and advanced the pipette until it almost reached the opposite side of the zygote’s cortex. Approximately 5 pg of px330 was injected into each zygote. Injected zygotes were cultured in KSOM medium (#MR-121-D, MERCK, Darmstadt, Germany) at 37°C in a 5% CO_2_ incubator until the two-celled stage and were then transferred into pseudo-pregnant ICR mice to obtain the initial generation of genome-edited mice, F_0_. The protocols were approved by the Institutional Animal Care and Use Committee of Kyushu University.

### Surveyor nuclease assay

F_0_ mice were screened by surveyor nuclease assay using a Surveyor Mutation Detection Kit (#706020, Integrated DNA Technologies, Coralville, IA, U.S.A.) according to the manufacturer’s instructions. The products were assayed by agarose gel electrophoresis for 25 min at 135 V.

### Establishment of the mouse strain

The F_0_ generation mice determined to be positive by the surveyor nuclease assay were bred with wildtype C57BL/6J to produce the F_1_ mice, which were identified by PCR and sequencing. Male infertility has been suggested in *Ddhd1*(−/−) mice; because of impaired sperm mobility, we maintained the strain by crossing heterozygotes (*Ddhd1*[+/−]) with wildtypes. We generated homozygous KO mice (*Ddhd1*[−/−]) by crossing heterozygous (*Ddhd1*[+/−]) male and female siblings.

### DNA preparation

We collected tails from 2 weeks of age mice for genotyping. The tails were immersed in 50 μM NaOH at 95°C for 10 min and centrifuged at 12000 rpm for 15 min. We performed PCR using the supernatant fluid as a PCR template.

### Sanger sequencing

Exon 2 of *Ddhd1* was sequenced by direct sequencing using the primers Mouse_Ddhd1_forward and Mouse_Ddhd1_reverse (Supplementary Table S7). The PCR conditions consisted of 35 cycles at 94°C for 30 s, 58°C for 30 s and 72°C for 30 s. Template DNA for the sequencing reaction was prepared by the enzymatic reaction with 0.1 U of thermosensitive alkaline phosphatase (#10699730, Thermo Fisher Scientific, Waltham, MA, U.S.A.) and 1.2 U of exonuclease I (#M0293S, New England Biolabs, Ipswich, MA, U.S.A.) at 37°C for 30 min, followed by heat inactivation at 80°C for 15 min. The PCR products were then sequenced using the ABI PRISM Big Dye Terminator Cycle Sequencing Kit (v 3.1) (Applied Biosystems, Waltham, MA, U.S.A.) and the ABI PRISM 3130-Avant Genetic Analyzer (#4337456, Applied Biosystems, Waltham, MA, U.S.A.) according to the manufacturer’s protocol.

### FBA analysis

We allowed mice to walk on an elevated horizontal plastic beam (50′ × 5 cm) with 20 cm of the length and shot their videos from behind by iPhone (Apple, Cupertino, CA, U.S.A.). Once the mouse walked to the middle of plastic beam, it was brought back to the front with its tail until more than 20 steps were recorded. Angles at the toe-off positions of hind paws of was measured as FBA for the first 20 steps using single video frames at 14 months of age (*n*=3) and 24 months of age (*n*=2) awere examined. The data between *Ddhd1*(+/−) and *Ddhd1*(−/−) were analyzed using a two-tailed Student’s *t* test and the data between individuals were analyzed using a Tukey–Kramer test. Tukey–Kramer test was performed in R Studio (version 3.5.3).

### Animals used in tissue sampling

At 26 months of age, *Ddhd1*(+/−) and *Ddhd1*(−/−) (*n*=2 per group) were killed with cervical spine fracture dislocation without anesthesia. Cerebra were extracted from each mouse and immediately snap-frozen in liquid nitrogen for subsequent lipid and protein isolation. The cerebra samples for RNA-seq were immediately immersed in RNA*later* Solutions (#AM7021, Invitrogen, Carlsbad, CA, U.S.A.). We utilized all available animals (two mice each for *Ddhd1*(+/−) and *Ddhd1*(−/−)) after the long-term rearing of 26 months. Details of the mice used in FBA tests, lipidome analyses and RNA sequencing were described in Supplementary Table S8.

### Western blotting

Since the cerebrum has been reported to show the highest *Ddhd1* expression [8], we extracted total protein from cerebral tissues of 12-month-old *Ddhd1*(+/−) and *Ddhd1*(−/−) mice using T-PER Tissue Protein Extraction Reagent (#78510, Thermo Fisher Scientific, Waltham, MA, U.S.A.) according to the standard protocol. Rabbit anti-DDHD1, anti-COXIV and anti-rabbit IgG were obtained from Atlas antibodies (#HPA049870, Bromma, Sweden), Cell Signaling Technology (#4844, Danvers, MA, U.S.A.) and Southern Biotechnology Associates (#4055-05, Birmingham, AL, U.S.A.) respectively. Protein was transferred to nitrocellulose membrane using iBlot 2 DRY Blotting System (Invitrogen, Waltham, MA, U.S.A.). Antibody reaction was performed using iBind Western Systems (Invitrogen, Waltham, MA, U.S.A.) according to the manufacturer’s protocol. We detected chemiluminescence using SuperSignal West Dura Extended Duration Substrate (#34075, Thermo Fisher Scientific, Waltham, MA, U.S.A.) and LAS4000mini (GE Healthcare Life Science, Buckinghamshire, England). SeeBlue Plus2 Pre-Stained Standard (#LC5925, Thermo Fisher Scientific, Waltham, MA, U.S.A.) was used as a molecular weight marker.

### Lipid extraction

We performed lipid extraction from cerebra and cerebella (∼5 mg each) extracted from 26-month-old *Ddhd1*(+/−) and *Ddhd1*(−/−) (*n*=2 per group) using an acidic methanol extraction. In brief, frozen cerebra and cerebella tissues were individually ground with a ball mill at 20 Hz for 3 min (Model MM301, Retsch, Haan, Germany) and extracted with 1 ml of 20 mM acetic acid in methanol containing the internal standards: PI 15:0‒18:1 (d_7_), 240 pmol; LPI 17:1, 3400 pmol; PA 15:0‒18:1 (d_7_), 200 pmol; LPA 17:0, 1100 pmol. Samples were mixed vigorously by vortexing for 1 min followed by 5 min of sonication. The supernatant (700 μl) after centrifugation by 16000×***g*** for 5 min at 4°C was transferred to clean tubes. The extracted supernatants were dried under nitrogen and stored at −80°C until analysis. Prior to analysis, the dried sample was reconstituted in 200 μl of methanol/chloroform (1:1, vol/vol).

### Chemicals and reagents used in lipidome analysis

Ammonium acetate was obtained from Sigma–Aldrich (#73594, St. Louis, MO, U.S.A.). LC/MS-grade methanol and distilled water were purchased from Kanto Chemical Co. (#11307-2B, Tokyo, Japan). HPLC-grade chloroform was obtained from Kishida Chemical (#140-16013, Osaka, Japan). All synthetic lipid standards were purchased from Avanti Polar Lipids Inc. (Alabaster, AL, U.S.A.). Carbon dioxide (99.9% grade; Yoshida Sanso Co., Fukuoka, Japan) was used as the SFC mobile phase.

### SFC/MS/MS

The levels of PI, LPI, PA and LPA were quantified using SFC/QqQMS in multiple reaction mode, as described previously [14]. The SFC/MS/MS system is composed of a Shimadzu Nexera UC and a Shimadzu LCMS-8060 triple–quadrupole mass spectrometer equipped with an electrospray ionization ion source (Shimadzu Co., Kyoto, Japan). The SFC conditions were as follows: 2 μl injection volume; mobile phase (A), supercritical carbon dioxide; mobile phase (B) (modifier) and make-up pump solvent; methanol/water (95/5, v/v) with 0.1% (w/v) ammonium acetate; 1.0 ml.min^−1^ flow rate of mobile phase; 0.1 ml.min^−1^ flow rate of make-up pump; modifier gradient; 1% (B) (1 min), 1‒75% (B) (23 min), 75% (B) (2 min), 75‒1% (B) (0.1 min), 1% (B) (3.9 min); column, ACQUITY UPC2™ Torus diethylamine (DEA) (3.0 × 100 mm, 1.7 μm, Waters Co., Milford, MA, U.S.A.); 50°C column temperature; 10 MPa back pressure regulator; and 30 min analytical time. The QqQMS conditions were as follows: positive and negative polarity (polarity switching mode), 4.0 kV electrospray voltage for positive and −3.5 kV for negative, 3 l.min^−1^ nebulizing gas flow rate, 10 l.min^−1^ heating gas flow rate, 10 l.min^−1^ dry gas flow rate, 250°C desolvation temperature, 400°C heat block temperature, and 2.3 kV detector voltage. The MRM parameters were as follows: 2 ms dwell time, 2 ms pause time, and 15 ms polarity switching time. Other optimized MRM parameters for each lipid are shown in Supplementary Tables S2 and S3. All data were analyzed using a two-tailed Student’s *t* test.

### RNA sequencing

Total RNAs were extracted from the cerebra of both 26-month-old *Ddhd1*(+/−) and *Ddhd1*(−/−) mice (*n*=2 per group) using the RNeasy Mini Kit (Qiagen) according to the manufacturer’s protocol. cDNA libraries were constructed from the purified RNA using the SMARTer Universal Low Input RNA Kit for Sequencing (#634938, Clontech, Mountain View, CA) and a Low Input Library Prep Kit (v 2) (#634947, Clontech, Mountain View, CA). Single-read 50-bp sequencing was performed on the Illumina HiSeq 2500. To generate a heatmap, we used the ‘heatmap.2’ function of the gplots package in R software. This analysis was performed in R Studio (version 3.5.3).

### Detection of DEGs and GEA

Raw reads obtained from NGS were mapped to the reference genome (Mouse GRCm38/mm19) using TopHat. DEGs were identified using Cuffdiff to test the significance of the differential expression of genes based on fragments per kilobase of exon per million fragments mapped (FPKM). Finally, The DEGs were submitted to the Database for Annotation, Visualization and Integrated Discovery (DAVID, version 6.7, https://david.ncifcrf.gov/home.jsp) to analyze the enrichment of GO terms. GO terms showing the Benjamini probabilities < 0.05 were considered as significant difference.

### Real-time quantity PCR

The purified RNA extracted from the cerebra of both 12-month-old *Ddhd1*(+/−) and *Ddhd1*(−/−) mice (*n*=2 per group) was converted in each instance into cDNA using ReverTra Ace qPCR RT Master Mix with gDNA Remover (TOYOBO, Osaka, Japan). RT-qPCR were then performed with Power SYBR Green Master Mix (#4367659, Applied Biosystems, Waltham, MA) on the Applied Biosystems 7500 Real-Time PCR System (Applied Biosystems, Waltham, MA). Expression of *Gapdh* was also examined as an internal standard of mRNA expression. Primers are shown in Supplementary Table S7.

### Perfusion fixation and immunohistochemistry

We performed immunohistochemistry using spinal cords perfusion-fixed with 4% paraformaldehyde phosphate buffer solution (#163-20145, FUJIFILM, Tokyo, Japan) of *Ddhd1*(+/−) and *Ddhd1*(−/−) at 6 months of age. For ultrathin sectioning, *Ddhd1*(+/−) and *Ddhd1*(−/−) at 6 months of age were perfused with 4% paraformaldehyde phosphate buffer solution. The mice were anesthetized by intraperitoneal infection of mixed anesthesia (10 ml/kg) and killed by dissection of the right atrium. Mixed anesthesia was composed of medetomidine hydrochloride (0.3 mg/kg) (Kyoritsu Seiyaku, Tokyo, Japan), midazolam (4 mg/kg) (Teva Takeda Pharma Ltd., Nagoya, Japan) and butorphanol tartrate (5 mg/kg) (Meiji Seika Pharma Co, Ltd., Tokyo, Japan). Spinal cords from each mouse were embedded in paraffin and sliced using a cryostat to obtain tissue sections. Immunohistochemistry was performed using primary antibodies against NF. Anti-NF was obtained from Dako (#M0762, Santa Clara, CA, U.S.A.). Sections were incubated with primary antibodies overnight at 4°C. After rinsing, immunoreaction products were detected by the polymer immunocomplex method using an Histfine Simple Stain MAX PO(M) (#414321, Nichirei Biosciences, Tokyo, Japan). Immunoreactivity was visualized using 3,3′-diaminobenzidine (DAB) (#D006, Dojindo, Kumamoto, Japan) and specimens were lightly counterstained with Hematoxylin. Microscopic images were acquired by All-In-One Fluorescence Microscope BZ-X700 (KEYENCE, Osaka, Japan). The acquired images were analyzed using ImageJ software.

## Supplementary Material

Supplementary Figures S1-S7 and Tables S1-S8

## Data Availability

NGS data can be accessed from NCBI and DDBJ. RNA-seq data can be accessed in DRA009135 (SAMD00191356-00191359). The datasets generated during the current study are available from the corresponding author on reasonable request.

## Abbreviations

DEG: differentially expressed gene
FBA: foot–base angle
GEA: GO enrichment analysis
GO: gene ontology
GPCR: G protein-coupled receptor
GPR55: G-protein coupled receptor 55
ICR: Institute of Cancer Research
indel: insertion and/or deletion
KO: knockout
LPA: lysophosphatidic acid
LPI: lysophosphatidylinositol
NF: neurofilament
NGS: next generation sequencing
OMIM: Online Mendelian Inheritance in Man
PA: phosphatidic acid
PI: phosphatidylinositol
PLA_1_: phospholipase A_1_
PLA_2_: phospholipase A_2_
RT-qPCR: real-time quantitative PCR
SCA: spinocerebellar ataxia
SFC/QqQMS: supercritical fluid chromatography triple–quadrupole mass spectrometry
SPG: spastic paraplegia
SPG28: spastic paraplegia type 28
