# Supplementary material for: Ddhd1 knockout mouse as a model of locomotive and physiological abnormality in familial spastic paraplegia
Source: Biosci Rep. 2021 Feb 26;41(2):BSR20204171. doi: 10.1042/BSR20204171 (PMC7921290; doi:10.1042/BSR20204171)
Supplement: Supplementary Figures S1-S7 and Tables S1-S8 [file BSR-2020-4171_supp.pdf]

Supplementary Figure S1

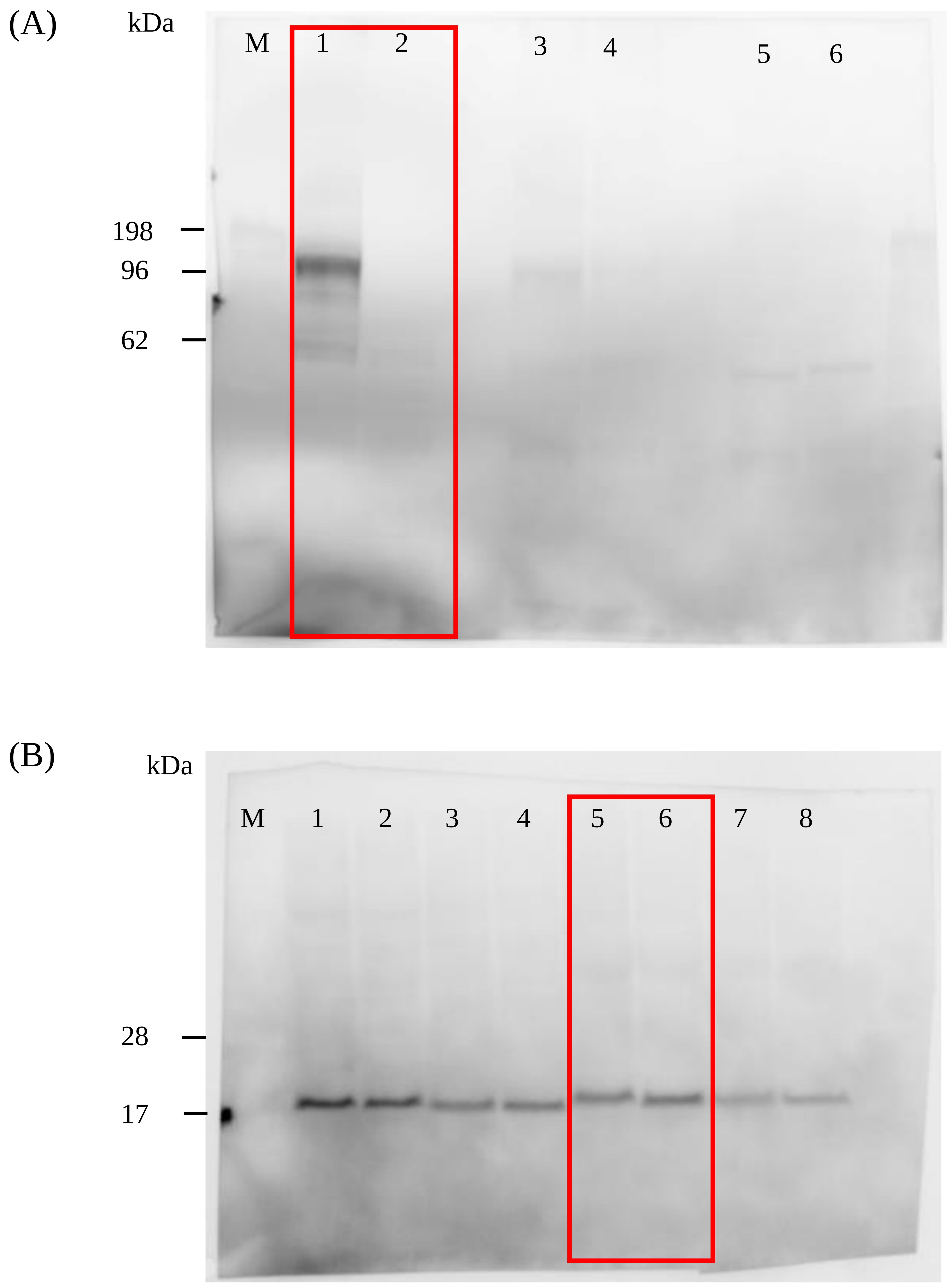

Figure S1.
(A) Western blotting with an antibody against Ddhd1. SeeBlue Plus2 Pre-Stained Protein Standard (Life Technologies) was used as a protein molecular weight marker. Lane M: Marker, Lane 1: *Ddhd1*(+/−) cerebrum, Lane 2: *Ddhd1*(−/−) cerebrum, Lane 3: *Ddhd1*(+/−) cerebrum diluted by 2 times from lane 1, Lane 4: *Ddhd1*(−/−) cerebrum diluted by 2 times from lane 2, Lane 5: *Ddhd1*(+/−) cerebrum diluted by 4 times from lane 1, *Ddhd1*(−/−) cerebrum diluted by 4 times from lane 1. (B) Western blotting with an antibody against COXIV as a loading control. Lane M: Marker, Lane 1 *Ddhd1*(+/−) testis, Lane 2: *Ddhd1*(−/−) testis, Lane 3: *Ddhd1*(+/−) testis diluted by 2 times from lane 1, Lane 4: *Ddhd1*(−/−) testis diluted 2 times from Lane 2, Lane 5: *Ddhd1*(+/−) cerebrum, Lane 6: *Ddhd1*(−/−) cerebrum, Lane 7: *Ddhd1*(+/−) cerebrum diluted 2 times from lane 5. Lane 8: *Ddhd1*(−/−) cerebrum diluted 2 times from lane 6. Both images were obtained in 4sec exposure time. The regions surrounded by red frames were used for Figure 1d.

Supplementary Figure S2

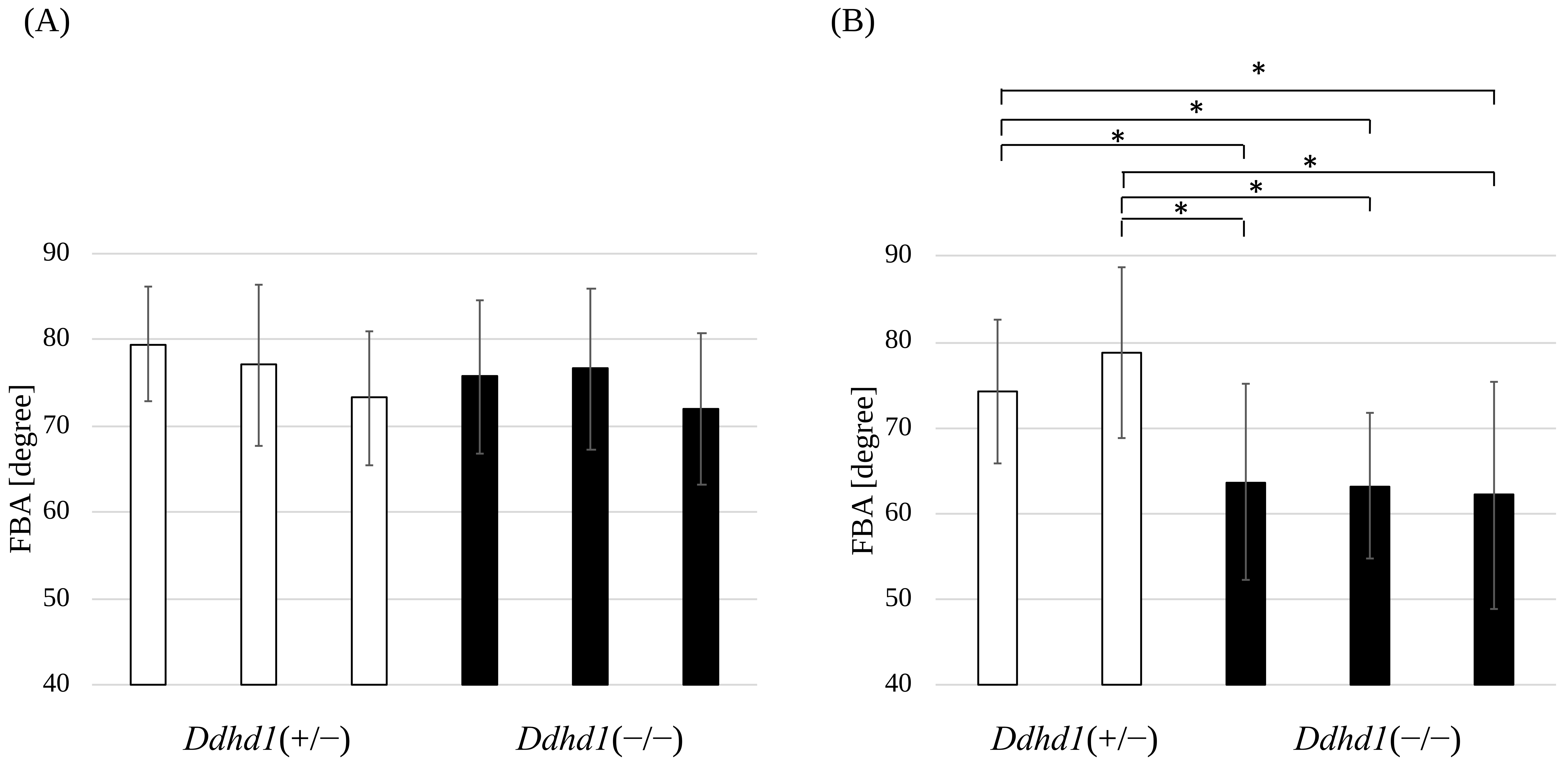

Figure S2. Foot–base angle between individuals. (A) The FBA of mice at the 14 months of age. There were no significant differences between any of the individuals (n = 3). (B) The FBA of mice at 24 months of age. We observed consistent decrease of FBA in *Ddhd1*(−/−) mice (n = 3) compared with that of *Ddhd1*(+/−) mice (n = 2), which is statistically significant (*p < 0.05). Error bars represent the mean ± SD. All data were analyzed using a Tukey-Kramer test.

Supplementary Figure S3

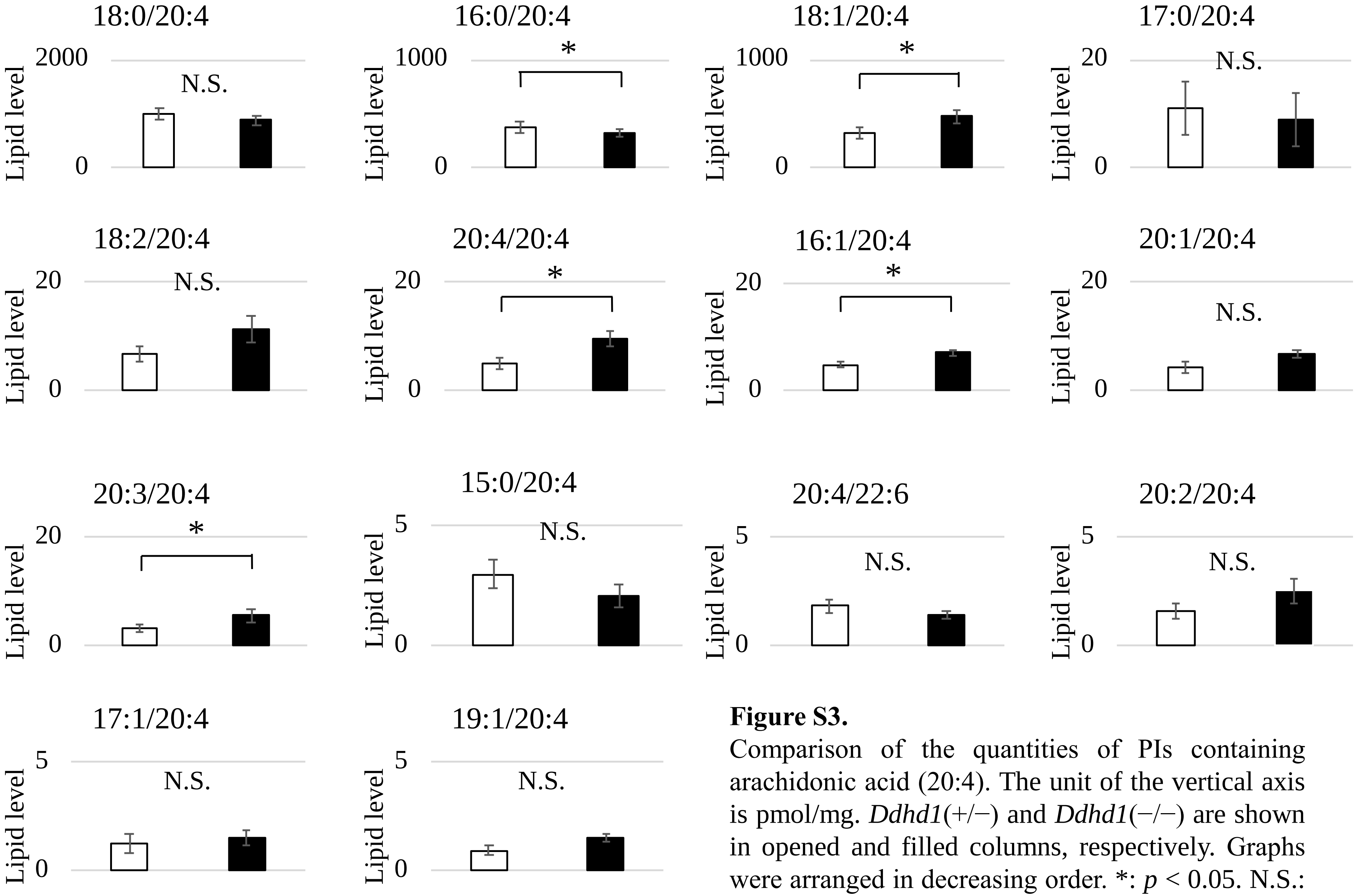

Figure S3.
Comparison of the quantities of PIs containing arachidonic acid (20:4). The unit of the vertical axis is pmol/mg. *Ddhd1*(+/−) and *Ddhd1*(−/−) are shown in opened and filled columns, respectively. Graphs were arranged in decreasing order. *: $p < 0.05$. N.S.: not significant. (a two-tailed Student t test).

Supplementary Figure S4

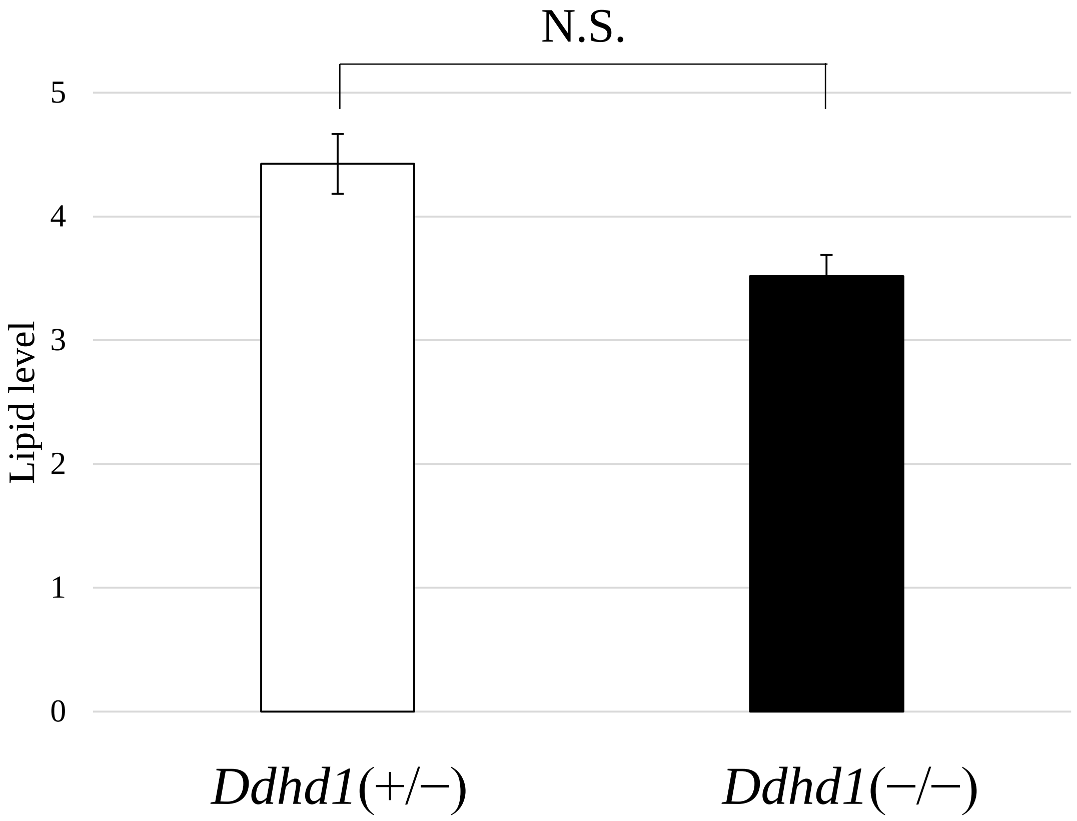

Figure S4. The amount of LPI 20:4 (*sn*-2) in mouse cerebella.
The unit of the vertical axis is pmol/mg. Error bars represent the mean ± SD. For 26-months of age *Ddhd1*(+/−) (*n* = 2) versus *Ddhd*1(−/−) (*n* = 2), N.S.: not significant. All data were analyzed using a two-tailed Student's t test.

Supplementary Figure S5

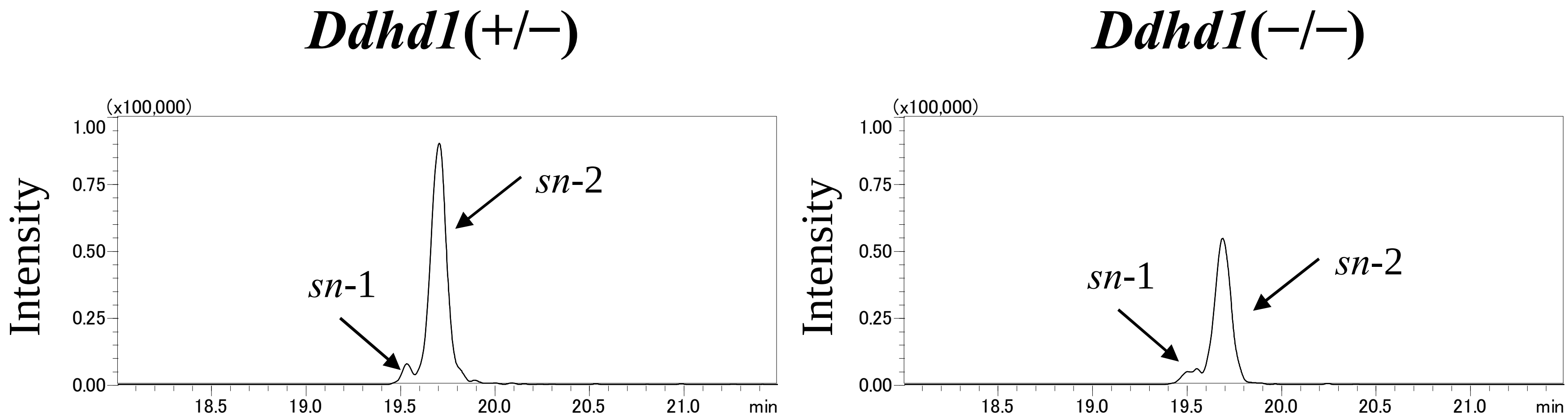

Figure S5.
SFC/MS/MS chromatograms of detected LPI 20:4 in mouse cerebra extracts. Retention times of LPI 20:4 (*sn*-1) and LPI 20:4 (*sn*-2) were 19.55 and 19.70 minutes, respectively.

Supplementary Figure S6

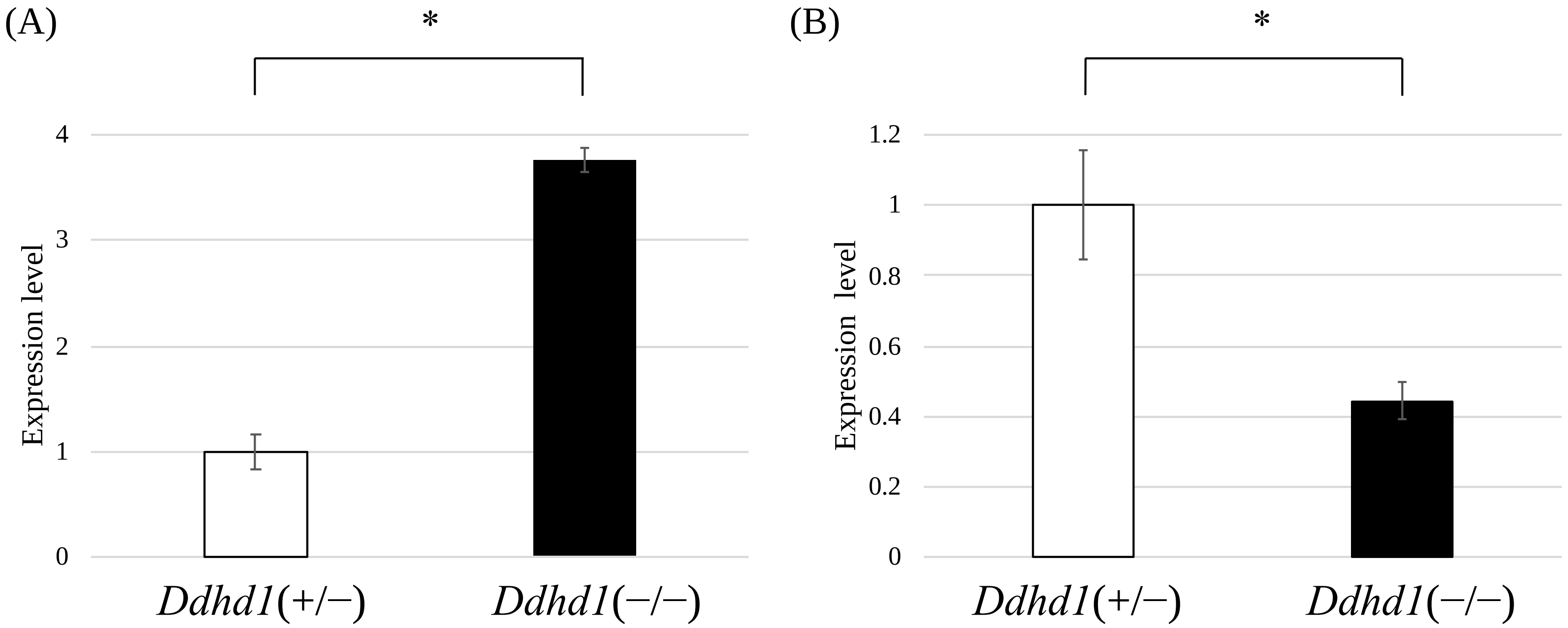

Figure S6. Quantitative determination of *Rtn4r* (A) and *Adra2a* (B) mRNA expression levels. The *Rtn4r* and *Adra2a* mRNA expression levels were quantified by real-time qPCR. Values represent mean ± SD normalized by Gapdh mRNA levels. *:$p < 0.05$

Supplementary Figure S7

(A) (B)

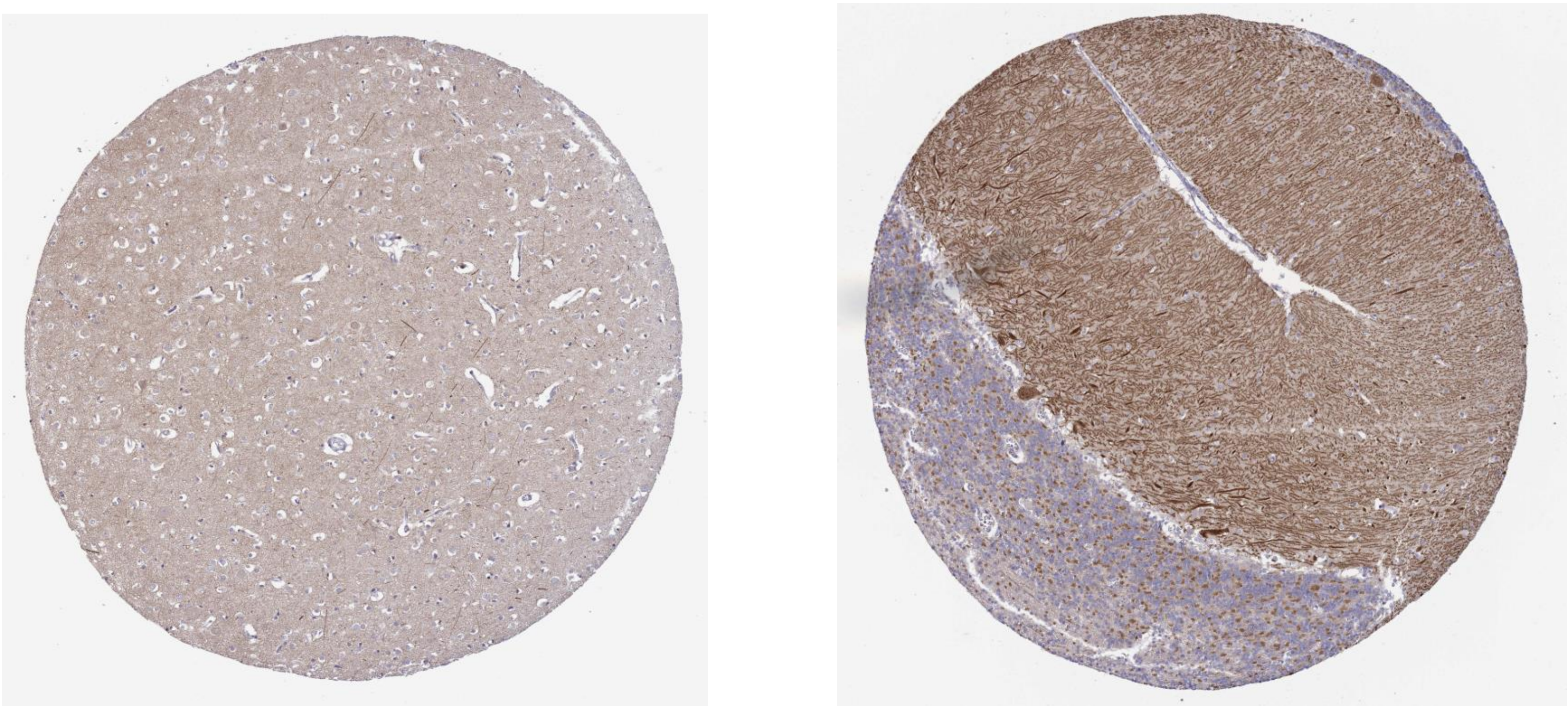

Figure S7. DDHD1 protein expression in human brain from The Human Protein Atlas database.

Highly DDHD1 protein expression is observed in human cerebrum (A) and cerebellum (B). Data courtesy of Protein Atlas.org.

Supplementary Table S1. Individual differences in FBA.

(A) 14 months of age

	Ddhd1 (+/−)_Individual 1				
Ddhd1 (+/−)_Individual 2	0.94	*Ddhd1* (+/−)_Individual 2			
Ddhd1 (+/−)_Individual 3	0.20	0.72	*Ddhd1* (+/−)_Individual 3		
Ddhd1 (−/−)_Individual 1	0.90	>0.99	0.94	*Ddhd1* (−/−)_Individual 1	
Ddhd1 (−/−)_Individual 2	0.81	>0.99	0.81	>0.99	*Ddhd1* (−/−)_Individual 2
Ddhd1 (−/−)_Individual 3	0.06	0.41	>0.99	0.72	0.51

(B) 24 months of age

	Ddhd1 (+/−)_Individual 1			
Ddhd1 (+/−)_Individual 2	0.64	*Ddhd1* (+/−)_Individual 2		
Ddhd1 (−/−)_Individual 1	1.8×10^{-2}*	5.0×10^{-5}*	*Ddhd1* (−/−)_Individual 1	
Ddhd1 (−/−)_Individual 2	1.1×10^{-2}*	8.5×10^{-5}*	>0.99	*Ddhd1* (−/−)_Individual 2
Ddhd1 (−/−)_Individual 3	4.3×10^{-3}*	2.6×10^{-5}*	>0.99	>0.99

Individual difference of FBA in mice at 14 months of age (A) and at 24 months of age (B). FBA was examined by Tukey-Kramer test with 20 strides per animal. *$p < 0.05$

Supplementary Table S2. PIs and LPIs detected in mouse cerebera.

		Formula	Exact mass	Precursor-ion	Product-ion	MRM transition	Retention time (min)	*Ddhd1* (+/−)					*Ddhd1* (−/−)					
								Individual 1		Individual 2		Average (pmol/mg)	Individual 1		Individual 2		Average (pmol/mg)	
								Exp 1 (pmol/mg)	Exp 2 (pmol/mg)	Exp 1 (pmol/mg)	Exp 2 (pmol/mg)		Exp 1 (pmol/mg)	Exp 2 (pmol/mg)	Exp 1 (pmol/mg)	Exp 2 (pmol/mg)		*p* -value
	PI 15:0/16:0	C40H77O13P1	796.5102	[M - H]-	[Acyl FA - H]-	795.5>241.2, 795.5>255.25	16.36	1.02	1.12	2.80	0.667	1.40	0.988	1.57	0.797	0.615	0.993	0.449
	PI 15:0/18:1	C42H79O13P1	822.5258	[M - H]-	[Acyl FA - H]-	821.5>241.2, 821.5>281.25	16.37	2.52	2.80	7.04	3.38	3.94	2.28	3.64	1.30	1.18	2.10	0.355
	PI 15:0/20:1	C44H83O13P1	850.5571	[M - H]-	[Acyl FA - H]-	849.55>309.3, 849.55>241.2	16.30	0.457	0.370	1.55	0.550	0.733	0.233	0.332	0.177	0.352	0.273	0.284
	PI 15:0/20:4	C44H77O13P1	844.5102	[M - H]-	[Acyl FA - H]-	843.5>241.2, 843.5>303.25	16.56	2.74	3.80	2.48	2.94	2.99	1.60	2.09	2.70	1.80	2.05	0.112
	PI 16:0/18:0	C43H83O13P1	838.5571	[M - H]-	[Acyl FA - H]-	837.55>283.25, 837.55>255.25	17.42	11.0	14.6	21.0	16.2	15.7	11.1	12.6	17.9	12.0	13.4	0.557
	PI 16:0/18:1	C43H81O13P1	836.5415	[M - H]-	[Acyl FA - H]-	835.55>281.25, 835.55>255.25	16.83	33.7	43.4	66.1	46.2	47.3	35.6	47.0	54.9	40.2	44.4	0.784
	PI 16:0/18:2	C43H79O13P1	834.5258	[M - H]-	[Acyl FA - H]-	833.5>279.25, 833.5>255.25	16.93	2.09	2.50	3.83	2.88	2.83	1.75	1.62	3.17	2.10	2.16	0.449
	PI 16:0/20:1	C45H85O13P1	864.5728	[M - H]-	[Acyl FA - H]-	863.55>309.3, 863.55>255.25	16.72	1.70	2.15	3.92	2.62	2.60	2.42	2.35	3.00	2.04	2.45	0.850
	PI 16:0/20:2	C45H83O13P1	862.5571	[M - H]-	[Acyl FA - H]-	861.55>255.25, 861.55>307.25	16.77	0.702	1.22	1.97	0.808	1.18	1.10	1.05	1.42	0.983	1.14	0.882
	PI 16:0/20:3	C45H81O13P1	860.5415	[M - H]-	[Acyl FA - H]-	859.55>255.25, 859.55>305.25	16.98	19.5	30.1	26.6	25.3	25.4	20.4	24.8	25.4	20.5	22.8	0.0495
	PI 16:0/20:4	C45H79O13P1	858.5258	[M - H]-	[Acyl FA - H]-	857.5>255.25, 857.5>303.25	16.97	318	434	361	375	372	290	357	363	291	325	0.00843
	PI 16:0/20:5	C45H77O13P1	856.5102	[M - H]-	[Acyl FA - H]-	855.5>255.25, 855.5>301.2	17.07	4.88	6.58	6.85	7.18	6.37	4.80	6.01	5.09	5.59	5.37	0.260
	PI 16:0/22:4	C47H83O13P1	886.5571	[M - H]-	[Acyl FA - H]-	885.55>255.25, 885.55>331.25	16.88	3.31	4.22	4.66	4.45	4.16	3.97	4.28	3.35	3.24	3.71	0.514
	PI 16:0/22:5	C47H81O13P1	884.5415	[M - H]-	[Acyl FA - H]-	883.55>255.25, 883.55>329.25	17.08	5.77	6.92	8.00	6.44	6.78	4.87	6.13	6.84	4.09	5.48	0.0972
	PI 16:0/22:6	C47H79O13P1	882.5258	[M - H]-	[Acyl FA - H]-	881.5>255.25, 881.5>327.25	17.06	53.0	71.4	87.7	64.6	69.2	52.5	67.9	75.4	47.2	60.7	0.353
	PI 16:0/16:1	C41H77O13P1	808.5102	[M - H]-	[Acyl FA - H]-	807.5>255.25, 807.5>253.2	17.61	2.15	1.59	4.00	2.50	2.56	1.39	1.32	1.82	2.10	1.66	0.354
	PI 16:1/18:1	C43H79O13P1	834.5258	[M - H]-	[Acyl FA - H]-	833.5>281.25, 833.5>253.2	17.60	1.73	2.21	4.35	2.94	2.81	2.83	3.51	4.29	3.68	3.58	0.495
	PI 16:1/20:4	C45H77O13P1	856.5102	[M - H]-	[Acyl FA - H]-	855.5>253.2, 855.5>303.25	17.09	4.42	4.83	4.21	5.72	4.80	6.57	7.63	7.48	6.68	7.09	0.00546
	PI 17:0/18:1	C44H83O13P1	850.5571	[M - H]-	[Acyl FA - H]-	849.55>281.25, 849.55>269.25	16.77	0.232	0.330	0.547	0.540	0.413	0.320	0.449	0.597	0.339	0.428	0.924
	PI 17:0/20:4	C46H81O13P1	872.5415	[M - H]-	[Acyl FA - H]-	871.55>269.25, 871.55>303.25	16.91	7.45	10.2	14.1	12.2	11.0	8.65	8.78	9.93	8.53	8.97	0.451
	PI 17:0/22:6	C48H81O13P1	896.5415	[M - H]-	[Acyl FA - H]-	895.55>269.25, 895.55>327.25	17.03	0.652	0.684	1.47	0.640	0.860	0.627	1.07	0.914	0.510	0.780	0.737
	PI 17:1/20:4	C46H79O13P1	870.5258	[M - H]-	[Acyl FA - H]-	869.5>303.25, 869.5>267.25	17.03	0.906	0.832	1.75	1.50	1.25	1.20	2.05	1.51	1.45	1.55	0.511
	PI 18:0/18:0	C45H87O13P1	866.5884	[M - H]-	[Acyl FA - H]-	865.6>283.25	16.63	3.25	3.59	4.82	3.07	3.68	1.96	2.79	3.30	2.90	2.74	0.169
	PI 18:0/20:0	C47H91O13P1	894.6197	[M - H]-	[Acyl FA - H]-	893.6>283.25, 893.6>311.3	16.52	1.14	1.50	2.27	1.42	1.58	1.27	1.69	1.94	1.49	1.60	0.963
PIs	PI 18:0/20:1	C47H89O13P1	892.6041	[M - H]-	[Acyl FA - H]-	891.6>309.3, 891.6>283.25	16.64	4.55	6.11	8.74	7.55	6.74	4.82	4.45	7.35	5.08	5.43	0.502
	PI 18:0/20:3	C47H85O13P1	888.5728	[M - H]-	[Acyl FA - H]-	887.55>283.25, 887.55>305.25	16.85	58.2	78.0	63.9	66.6	66.7	51.3	64.4	60.6	51.2	56.9	0.0293
	PI 18:0/20:4	C47H83O13P1	886.5571	[M - H]-	[Acyl FA - H]-	885.55>283.25, 885.55>303.25	16.85	904	1112	935	1043	999	823	1018	883	824	887	0.0842
	PI 18:0/20:5	C47H81O13P1	884.5415	[M - H]-	[Acyl FA - H]-	883.55>283.25, 883.55>301.2	16.97	6.28	8.04	8.73	9.11	8.04	4.83	5.10	5.78	5.99	5.43	0.119
	PI 18:0/22:1	C49H93O13P1	920.6354	[M - H]-	[Acyl FA - H]-	919.65>337.3, 919.65>283.25	16.55	1.60	1.51	2.40	1.37	1.72	1.06	1.93	1.83	1.47	1.57	0.503
	PI 18:0/22:4	C49H87O13P1	914.5884	[M - H]-	[Acyl FA - H]-	913.6>283.25, 913.6>331.25	16.75	5.20	6.19	6.10	5.36	5.71	3.97	4.95	4.70	4.23	4.46	0.000200
	PI 18:0/22:5	C49H85O13P1	912.5728	[M - H]-	[Acyl FA - H]-	911.55>283.25, 911.55>329.25	16.96	4.17	3.93	7.03	5.26	5.10	2.57	3.12	3.61	2.82	3.03	0.191
	PI 18:0/22:6	C49H83O13P1	910.5571	[M - H]-	[Acyl FA - H]-	909.55>283.25, 909.55>327.25	16.94	37.7	49.8	76.7	47.9	53.0	33.8	37.8	44.2	29.3	36.3	0.213
	PI 18:0/18:1	C45H85O13P1	864.5728	[M - H]-	[Acyl FA - H]-	863.55>283.25, 863.55>281.25	16.69	44.7	55.8	74.5	60.9	59.0	28.0	35.8	44.3	36.3	36.1	0.142
	PI 18:1/18:1	C45H83O13P1	862.5571	[M - H]-	[Acyl FA - H]-	861.55>281.25	16.82	7.46	9.68	18.0	12.4	11.9	14.6	19.9	19.6	17.6	17.9	0.215
	PI 18:1/20:1	C47H87O13P1	890.5884	[M - H]-	[Acyl FA - H]-	889.6>281.25, 889.6>309.3	16.75	1.28	1.65	3.00	2.11	2.01	2.62	3.23	4.14	2.96	3.24	0.190
	PI 18:1/20:3	C47H83O13P1	886.5571	[M - H]-	[Acyl FA - H]-	885.55>281.25, 885.55>305.25	16.97	18.8	24.4	20.6	20.6	21.1	30.9	37.4	34.3	29.7	33.1	0.00923
	PI 18:1/20:4	C47H81O13P1	884.5415	[M - H]-	[Acyl FA - H]-	883.55>281.25, 883.55>303.25	16.97	278	380	294	346	324	436	551	485	425	474	0.0171
	PI 18:1/22:1	C49H91O13P1	918.6197	[M - H]-	[Acyl FA - H]-	917.6>281.25, 917.6>337.3	16.65	0.447	0.262	1.02	0.672	0.600	1.04	0.801	0.889	0.789	0.880	0.376
	PI 18:1/22:4	C49H85O13P1	912.5728	[M - H]-	[Acyl FA - H]-	911.55>281.25, 911.55>331.25	16.88	0.989	1.29	2.37	1.34	1.50	2.62	3.01	4.20	2.70	3.13	0.0759
	PI 18:1/22:6	C49H81O13P1	908.5415	[M - H]-	[Acyl FA - H]-	907.55>281.25, 907.55>327.25	17.07	7.41	10.3	13.7	12.0	10.8	12.8	15.0	16.7	10.7	13.8	0.282
	PI 18:0/18:2	C45H83O13P1	862.5571	[M - H]-	[Acyl FA - H]-	861.55>283.25, 861.55>279.25	16.80	2.42	2.93	5.41	5.10	3.97	2.62	2.87	4.52	2.51	3.13	0.598
	PI 18:1/18:2	C45H81O13P1	860.5415	[M - H]-	[Acyl FA - H]-	859.55>281.25, 859.55>279.25	16.92	1.29	1.75	2.94	2.56	2.14	2.81	3.05	3.45	2.56	2.97	0.309
	PI 18:2/20:4	C47H79O13P1	882.5258	[M - H]-	[Acyl FA - H]-	881.5>279.25, 881.5>303.25	17.09	4.84	7.45	6.28	8.40	6.74	8.48	10.5	14.3	11.9	11.3	0.138
	PI 19:1/20:4	C48H83O13P1	898.5571	[M - H]-	[Acyl FA - H]-	897.55>295.25, 897.55>303.25	16.92	0.695	0.807	1.19	1.09	0.948	1.24	1.56	1.66	1.52	1.50	0.125
	PI 20:1/20:4	C49H85O13P1	912.5728	[M - H]-	[Acyl FA - H]-	911.55>309.3, 911.55>303.25	16.86	2.71	4.12	5.07	4.55	4.11	6.13	6.69	7.78	6.10	6.68	0.0753
	PI 20:2/20:4	C49H83O13P1	910.5571	[M - H]-	[Acyl FA - H]-	909.55>307.25, 909.55>303.25	16.93	1.71	1.73	1.10	1.98	1.63	2.30	2.25	3.44	2.23	2.56	0.0880
	PI 20:3/20:4	C49H81O13P1	908.5415	[M - H]-	[Acyl FA - H]-	907.55>305.25, 907.55>303.25	17.07	3.17	3.80	2.14	3.96	3.27	4.99	7.16	5.89	4.50	5.64	0.0404
	PI 20:4/20:4	C49H79O13P1	906.5258	[M - H]-	[Acyl FA - H]-	905.5>303.25	17.13	4.62	5.65	3.75	5.75	4.94	8.62	10.8	10.6	8.03	9.51	0.00373
	PI 20:4/22:6	C51H79O13P1	930.5258	[M - H]-	[Acyl FA - H]-	929.5>303.25, 929.5>327.25	17.22	1.47	1.91	1.84	2.16	1.85	1.28	1.43	1.72	1.28	1.43	0.135
	Total							1886	2430	2209	2268	2198	1951	2420	2270	1951	2148	0.457
	LPI 16:0 (*sn* -1)	C25H49O12P1	572.2962	[M - H]-	[Acyl FA - H]-	571.3>255.25	19.34	8.74	9.04	9.14	8.32	8.81	7.56	7.45	8.24	6.31	7.39	0.00959
	LPI 18:0 (*sn* -1)	C27H53O12P1	600.3275	[M - H]-	[Acyl FA - H]-	599.3>283.25	19.14	34.0	37.3	32.0	34.7	34.5	30.76	40.33	32.05	27.01	32.5	0.607
	LPI 18:1 (*sn* -1)	C27H51O12P1	598.3118	[M - H]-	[Acyl FA - H]-	597.3 > 281.25	19.30	5.65	7.14	5.91	6.81	6.38	7.20	9.03	9.42	7.85	8.38	0.0166
LPIs	LPI 18:1 (*sn* -2)	C27H51O12P1	598.3118	[M - H]-	[Acyl FA - H]-	597.3 > 281.25	19.44	0.432	0.215	0.744	0.399	0.448	0.22	0.60	0.94	0.38	0.535	0.667
	LPI 20:4 (*sn* -1)	C29H49O12P1	620.2962	[M - H]-	[Acyl FA - H]-	619.3 > 303.25	19.55	0.751	0.707	0.768	0.408	0.660	0.39	0.42	0.35	0.40	0.390	0.0637
	LPI 20:4 (*sn* -2)	C29H49O12P1	620.2962	[M - H]-	[Acyl FA - H]-	619.3 > 303.25	19.70	6.11	7.66	5.86	6.74	6.59	3.47	5.51	3.77	3.60	4.09	0.0373
	Total							55.6	62.0	54.4	57.3	57.3	49.60	63.34	54.77	45.55	53.5	0.393

Two strains were examined in two mice each with two technical replicates.

Supplementary Table S3. PAs and LPAs detected in mouse cerebera.

		Formula	Exact Mass	Precursor-ion	Product-ion	MRM transition	Retention time (min)	*Ddhd1* (+/−)					*Ddhd1* (−/−)					
								Individual 1		Individual 2		Average (pmol/mg)	Individual 1		Individual 2		Average (pmol/mg)	
								Exp 1 (pmol/mg)	Exp 2 (pmol/mg)	Exp 1 (pmol/mg)	Exp 2 (pmol/mg)		Exp 1 (pmol/mg)	Exp 2 (pmol/mg)	Exp 1 (pmol/mg)	Exp 2 (pmol/mg)		*p* -value
	PA 16:0/16:0	C35H69O8P1	648.4730058	[M - H]-	[Acyl FA - H]-	647.45>255.25	16.67	20.7	23.1	35.0	26.9	26.4	26.7	30.7	19.7	19.1	24.0	0.749
	PA 16:0/18:0	C37H73O8P1	676.5043059	[M - H]-	[Acyl FA - H]-	675.5>283.25, 675.5>255.25	16.51	24.9	27.2	48.6	30.3	32.8	39.8	49.4	24.2	27.1	35.1	0.857
	PA 16:0/18:1	C37H71O8P1	674.4886559	[M - H]-	[Acyl FA - H]-	673.5>255.25, 673.5>281.25	16.60	277	282	565	326	363	470	556	276	281	396	0.839
	PA 16:0/20:0	C39H77O8P1	704.5356061	[M - H]-	[Acyl FA - H]-	703.55>311.3, 703.55>255.25	16.38	3.27	3.56	8.61	3.45	4.72	5.94	7.80	2.26	4.05	5.01	0.910
	PA 16:0/20:1	C39H75O8P1	702.519956	[M - H]-	[Acyl FA - H]-	701.5>255.25, 701.5>309.3	16.46	18.8	20.2	49.1	24.0	28.0	38.4	48.2	22.5	25.6	33.7	0.702
	PA 16:0/20:3	C39H71O8P1	698.4886559	[M - H]-	[Acyl FA - H]-	697.5>255.25, 697.5>305.25	16.80	3.69	3.91	7.08	3.48	4.54	6.19	8.64	3.94	4.37	5.79	0.558
	PA 16:0/20:4	C39H69O8P1	696.4730058	[M - H]-	[Acyl FA - H]-	695.45>255.25, 695.45>303.25	16.85	30.2	36.7	57.9	36.2	40.2	45.7	61.8	30.1	31.8	42.4	0.888
	PA 16:0/22:1	C41H79O8P1	730.5512561	[M - H]-	[Acyl FA - H]-	729.55>337.3, 729.55>255.25	16.39	1.38	0.88	2.62	1.76	1.66	1.84	2.48	1.07	1.68	1.77	0.884
	PA 16:0/22:4	C41H73O8P1	724.5043059	[M - H]-	[Acyl FA - H]-	723.5>331.25, 723.5>255.25	16.66	6.16	5.05	8.20	4.97	6.09	7.11	8.77	5.74	6.00	6.90	0.552
	PA 16:0/22:6	C41H69O8P1	720.4730058	[M - H]-	[Acyl FA - H]-	719.45>255.25, 719.45>327.25	16.99	8.71	8.68	15.1	10.2	10.7	8.93	11.8	9.72	7.42	9.48	0.639
	PA 16:0/24:0	C43H85O8P1	760.5982063	[M - H]-	[Acyl FA - H]-	759.6>255.25, 759.6>367.35	16.04	0.97	1.11	2.07	0.79	1.23	2.45	3.61	0.41	1.08	1.88	0.631
	PA 16:0/24:1	C43H83O8P1	758.5825562	[M - H]-	[Acyl FA - H]-	757.6>365.35, 757.6>255.25	16.27	1.60	1.03	2.79	1.65	1.77	2.60	1.91	0.63	1.21	1.59	0.846
	PA 16:1/16:0	C35H67O8P1	646.4573557	[M - H]-	[Acyl FA - H]-	645.45>255.25, 645.45>253.2	16.77	8.63	11.0	19.8	12.9	13.1	11.7	17.6	7.20	7.12	10.9	0.705
	PA 16:1/18:0	C37H71O8P1	674.4886559	[M - H]-	[Acyl FA - H]-	673.5>283.25, 673.5>253.2	16.56	6.69	7.81	16.0	9.74	10.1	7.14	10.5	6.24	6.01	7.48	0.496
	PA 16:1/18:1	C37H69O8P1	672.4730058	[M - H]-	[Acyl FA - H]-	671.45>281.25, 671.45>253.2	16.80	3.95	4.75	11.6	6.85	6.79	8.44	10.6	4.65	4.70	7.10	0.937
	PA 17:0/18:1	C38H73O8P1	688.5043059	[M - H]-	[Acyl FA - H]-	687.5>269.25, 687.5>281.25	16.55	3.53	3.37	7.89	4.84	4.91	6.14	8.85	4.56	3.57	5.78	0.735
	PA 18:0/18:0	C39H77O8P1	704.5356061	[M - H]-	[Acyl FA - H]-	703.55>283.25	16.39	12.4	13.6	20.1	10.9	14.3	19.3	24.8	12.7	12.9	17.4	0.577
	PA 18:0/20:0	C41H81O8P1	732.5669062	[M - H]-	[Acyl FA - H]-	731.55>311.3, 731.55>283.25	16.32	3.06	2.31	4.28	3.06	3.18	4.91	4.97	2.92	2.92	3.93	0.571
	PA 18:0/20:1	C41H79O8P1	730.5512561	[M - H]-	[Acyl FA - H]-	729.55>283.25, 729.55>309.3	16.32	20.1	21.4	43.4	20.0	26.2	39.4	51.7	29.7	33.2	38.5	0.302
	PA 18:0/20:3	C41H75O8P1	726.519956	[M - H]-	[Acyl FA - H]-	725.5>283.25, 725.5>305.25	16.55	9.94	11.4	14.3	9.26	11.2	11.5	16.9	10.9	10.8	12.5	0.547
PAs	PA 18:0/20:4	C41H73O8P1	724.5043059	[M - H]-	[Acyl FA - H]-	723.5>283.25, 723.5>303.25	16.65	81.5	84.2	111.5	75.4	88.2	104.4	126.9	76.3	78.0	96.4	0.720
	PA 18:0/22:1	C43H83O8P1	758.5825562	[M - H]-	[Acyl FA - H]-	757.6>283.25, 757.6>337.3	16.20	0.753	0.784	1.77	1.08	1.10	1.25	1.67	1.26	0.731	1.23	0.777
	PA 18:0/22:4	C43H77O8P1	752.5356061	[M - H]-	[Acyl FA - H]-	751.55>283.25, 751.55>331.25	16.50	19.1	21.2	24.3	15.4	20.0	21.4	26.8	20.7	18.9	21.9	0.461
	PA 18:0/22:6	C43H73O8P1	748.5043059	[M - H]-	[Acyl FA - H]-	747.5>283.25, 747.5>327.25	17.44	25.1	34.3	31.7	30.6	30.4	32.4	33.8	32.7	26.5	31.3	0.678
	PA 18:0/24:0	C45H89O8P1	788.6295064	[M - H]-	[Acyl FA - H]-	787.6>283.25, 787.6>367.35	17.28	0.364	0.533	0.503	0.194	0.398	1.74	0.640	0.186	0.359	0.732	0.546
	PA 18:0/24:1	C45H87O8P1	786.6138564	[M - H]-	[Acyl FA - H]-	785.6>283.25, 785.6>365.35	16.18	1.12	1.23	1.71	0.880	1.23	2.56	1.95	0.597	0.987	1.52	0.732
	PA 18:1/18:0	C39H75O8P1	702.519956	[M - H]-	[Acyl FA - H]-	701.5>283.25, 701.5>281.25	16.42	300	315	513	279	352	452	580	304	325	415	0.623
	PA 18:1/18:1	C39H73O8P1	700.5043059	[M - H]-	[Acyl FA - H]-	699.5>281.25	16.57	76.2	75.7	178	96.4	107	190	226	110	114	160	0.446
	PA 18:1/20:0	C41H79O8P1	730.5512561	[M - H]-	[Acyl FA - H]-	729.55>311.3, 729.55>281.25	16.29	8.92	10.0	14.2	8.40	10.4	13.1	18.1	8.89	10.0	12.5	0.574
	PA 18:1/20:1	C41H77O8P1	728.5356061	[M - H]-	[Acyl FA - H]-	727.55>309.3, 727.55>281.25	16.47	20.9	23.7	54.6	28.0	31.8	47.0	62.8	32.3	34.0	44.0	0.486
	PA 18:1/20:4	C41H71O8P1	722.4886559	[M - H]-	[Acyl FA - H]-	721.5>281.25, 721.5>303.25	16.81	14.1	15.5	32.1	18.1	19.9	32.9	45.3	21.6	23.7	30.9	0.377
	PA 18:1/21:0	C42H81O8P1	744.5669062	[M - H]-	[Acyl FA - H]-	743.55>325.3, 743.55>281.25	16.39	1.36	1.89	3.11	1.45	1.95	1.79	2.93	1.19	1.71	1.91	0.939
	PA 18:1/22:0	C43H83O8P1	758.5825562	[M - H]-	[Acyl FA - H]-	757.6>339.35, 757.6>281.25	16.17	9.67	10.2	14.0	7.79	10.4	12.2	15.5	10.0	10.3	12.0	0.494
	PA 18:1/22:1	C43H81O8P1	756.5669062	[M - H]-	[Acyl FA - H]-	755.55>337.3, 755.55>281.25	16.33	8.00	7.61	14.9	8.84	9.83	12.1	12.6	8.84	9.49	10.8	0.752
	PA 18:1/23:0	C44H85O8P1	772.5982063	[M - H]-	[Acyl FA - H]-	771.6>353.35, 771.6>281.25	16.01	4.52	4.00	6.94	3.06	4.63	5.97	7.39	4.87	4.84	5.77	0.368
	PA 18:1/24:0	C45H87O8P1	786.6138564	[M - H]-	[Acyl FA - H]-	785.6>367.35, 785.6>281.25	15.96	7.81	8.42	11.8	5.74	8.44	13.8	14.6	6.92	9.20	11.1	0.475
	PA 18:1/24:1	C45H85O8P1	784.5982063	[M - H]-	[Acyl FA - H]-	783.6>281.25, 783.6>365.35	16.18	9.11	8.85	15.1	8.15	10.3	12.8	17.5	9.83	9.41	12.4	0.565
	PA 18:2/18:0	C39H73O8P1	700.5043059	[M - H]-	[Acyl FA - H]-	699.5>283.25, 699.5>279.25	16.57	8.20	9.63	17.0	11.4	11.6	12.2	15.5	9.83	10.3	12.0	0.915
	PA 18:2/18:1	C39H71O8P1	698.4886559	[M - H]-	[Acyl FA - H]-	697.5>281.25, 697.5>279.25	16.74	1.95	2.10	6.11	4.55	3.68	5.64	7.81	2.89	3.72	5.02	0.631
	PA 20:1/20:1	C43H81O8P1	756.5669062	[M - H]-	[Acyl FA - H]-	755.55>309.3	16.36	0.865	0.586	2.70	1.67	1.45	1.95	2.80	1.98	1.77	2.12	0.477
	PA 20:4/20:1	C43H75O8P1	750.519956	[M - H]-	[Acyl FA - H]-	749.5>303.25, 749.5>309.3	16.69	1.86	1.97	4.75	2.52	2.77	4.54	6.96	2.76	4.43	4.67	0.302
	Total							1067	1127	1999	1156	1337	1746	2165	1143	1189	1561	0.677
	LPA 14:1	C17H33O7P1	380.19639	[M - H]-	[C3H6O5P]-	379.2>153	20.83	0.073	0.108	0.066	0.065	0.078	0.148	0.133	0.085	0.053	0.105	0.552
	LPA 16:0	C19H39O7P1	410.2433402	[M - H]-	[C3H6O5P]-	409.25>153	21.78	0.079	0.135	0.226	0.123	0.141	0.157	0.172	0.110	0.107	0.137	0.938
	LPA 17:1	C20H39O7P1	422.2433402	[M - H]-	[C3H6O5P]-	421.25>153	21.45	0.787	0.862	0.436	0.622	0.677	0.619	0.679	0.802	0.699	0.700	0.895
	LPA 18:0	C21H43O7P1	438.2746403	[M - H]-	[C3H6O5P]-	437.25>153	21.45	0.618	0.802	0.796	0.701	0.729	0.733	0.787	0.603	0.581	0.676	0.601
	LPA 18:1	C21H41O7P1	436.2589903	[M - H]-	[C3H6O5P]-	435.25>153	21.83	0.154	0.135	0.348	0.186	0.206	0.204	0.275	0.151	0.193	0.206	1.00
	LPA 19:0	C22H45O7P1	452.2902904	[M - H]-	[C3H6O5P]-	451.3>153	21.33	0.297	0.266	0.319	0.324	0.302	0.289	0.329	0.234	0.273	0.281	0.614
	LPA 20:1	C23H45O7P1	464.2902904	[M - H]-	[C3H6O5P]-	463.3>153	21.41	0.030	0.042	0.090	0.045	0.052	0.051	0.064	0.029	0.052	0.049	0.898
LPAs	LPA 20:2	C23H43O7P1	462.2746403	[M - H]-	[C3H6O5P]-	461.25>153	21.51	0.088	0.079	0.077	0.061	0.076	0.080	0.070	0.087	0.053	0.073	0.677
	LPA 22:2	C25H47O7P1	490.3059405	[M - H]-	[C3H6O5P]-	489.3>153	21.52	0.177	0.166	0.162	0.174	0.170	0.186	0.187	0.140	0.143	0.164	0.828
	LPA 22:3	C25H45O7P1	488.2902904	[M - H]-	[C3H6O5P]-	487.3>153	21.58	0.131	0.132	0.122	0.142	0.132	0.097	0.147	0.122	0.137	0.125	0.225
	LPA 22:5	C25H41O7P1	484.2589903	[M - H]-	[C3H6O5P]-	483.25>153	21.60	0.436	0.451	0.429	0.502	0.454	0.448	0.468	0.409	0.416	0.435	0.534
	LPA 22:6	C25H39O7P1	482.2433402	[M - H]-	[C3H6O5P]-	481.25>153	21.47	0.330	0.396	0.374	0.339	0.360	0.403	0.334	0.314	0.307	0.339	0.560
	LPA 23:0	C26H53O7P1	508.3528907	[M - H]-	[C3H6O5P]-	507.35>153	21.65	0.137	0.125	0.157	0.136	0.139	0.156	0.132	0.122	0.117	0.132	0.675
	LPA 24:0	C27H55O7P1	522.3685407	[M - H]-	[C3H6O5P]-	521.35>153	21.53	3.84	4.52	5.08	6.02	4.86	3.89	4.39	6.21	5.34	4.96	0.938
	Total							7.17	8.21	8.68	9.44	8.38	7.46	8.16	9.42	8.47	8.38	0.998

Two strains were examined in two mice each with two technical replicates.

Supplementary Table S4. DEGs identified in *Ddhd1* KO mice.

Up or down	Gene	FPKM		Fold change
		Ddhd1 (+/−)	*Ddhd1* (−/−)	
Down	*Lrrc10b*	124	0.677	0.00548
	Drd2	44.2	0.627	0.0142
	Adora2a	36.9	0.578	0.0157
	Six3	10.0	0.175	0.0175
	Syndig1l	85.8	1.86	0.0216
	Rgs9	84.0	2.07	0.0246
	Serpina9	16.8	0.422	0.0252
	Drd1a	53.9	2.02	0.0375
	Tac1	220	12.9	0.0587
	Zfhx3	2.02	0.224	0.111
	Slc17a6	34.5	4.79	0.139
	Gpx6	5.18	0.00	-
	Gh	38.5	0.00	-
	Pax8	1.03	0.00	-
	Hoxb5	3.84	0.00	-
	Tfap2d	7.30	0.00	-
	Gm5868	1.26	0.00	-
	Hoxa5	1.36	0.00	-
	Rnf151	1.31	0.00	-
Up	*Rtn4r*	4.61	52.8	11.4
	Ccl21a,Gm1987	0.00	5.75	-
	Ccl21b,Gm10591	0.00	6.79	-

Expression levels were represented as FPKM. Fold change was calculated as $FPKM_{Ddhd1\ (-/-)}$ / $FPKM_{Ddhd1\ (+/-)}$.

Supplementary Table S5.
Expression level of *Ddhd1* .

Gene	FPKM		Fold change
	Ddhd1 (+/−)	*Ddhd1* (−/−)	
Ddhd1	1.84	0.823	0.448

Fold change was calculated as $\text{FPKM}_{\textit{Ddhd1}\ (-/-)}$ / $\text{FPKM}_{\textit{Ddhd1}\ (+/-)}$.

Suplementary Table 6. Oligonucleotides used in gene-targeting by the CRISPR/Cas9 system.

Primer name	Sequence
Mouse_CRISPR_target_S	caccAGTGGTTTATTGATGGGACG*
Mouse_CRISPR_target_AS	aaacCGTCCCATCAATAAACCACT*

*Overhangs for ligation into the *Bbs* I sites in px330 are shown is lower case.

Supplementary Table 8. Details of animals used in FBA tests, lipidome analyses, RNA sequencing and RT-qPCR.

Genotype	Individuals	sex	Age at death	Cause of death	Body weight when euthanized	Experiment
Ddhd1 (+/−)	Individual1	F	26 months	Euthanized	32.5 g	FBA test (14 months and 24 months), lipidome analysis, RNA sequencing
	Individual2	M	26 months	Euthanized	35.7 g	FBA test (14 months and 24 months), lipidome analysis, RNA sequencing
	Individual3	F	23 months	Unclear	—	FBA test (14 months)
Ddhd1 (−/−)	Individual1	F	26 months	Euthanized	30.6 g	FBA test (14 months and 24 months), lipidome analysis, RNA sequencing
	Individual2	M	26 months	Euthanized	36.7 g	FBA test (14 months and 24 months), lipidome analysis, RNA sequencing
	Individual3	M	24months	Unclear	—	FBA test (14 months and 24 months)

Six mice (n = 3 per group) were used for FBA test at 14 months of age. Five mice (*Ddhd1* (+/−): n = 2 and *Ddhd1* (−/−): n = 3) were used for FBA test at 24 months of age and four mice (n = 2 per group) were used for lipidome analysis and RNA sequencing at 26 months of age. F and M show female and male, respectively. *Ddhd1* (+/−) individual3 and *Ddhd1* (−/−) individual3 died in the cage before they turned 26 months of age.
